# Structure-property relationships of fluorinated carboxylic acid bioisosteres

**DOI:** 10.1016/j.bmcl.2023.129363

**Published:** 2023-06-08

**Authors:** Thibault Alle, Sipak Joyasawal, Killian Oukoloff, Keith Long, Zachary Owyang, Karol R. Francisco, Dominique Cahard, Donna M. Huryn, Carlo Ballatore

**Affiliations:** a Skaggs School of Pharmacy and Pharmaceutical Sciences, University of California, San Diego, 9500 Gilman Drive, La Jolla, CA 92093, United States; b Department of Pharmaceutical Sciences, University of Pittsburgh School of Pharmacy, 3501 Terrace St., Pittsburgh, PA 15261, United States; c CNRS, UMR 6014 COBRA, Normandie Université, 76821 Mont Saint Aignan, France

**Keywords:** Isosteric replacement, Acid bioisosteres, Fluorine, Structure-property relationship

## Abstract

Fluorinated alcohols and phenols are potentially useful as bioisosteres of the carboxylic acid functional group. To enable a direct comparison of the properties of fluorinated carboxylic acid surrogates with those of other commonly used, non-fluorinated bioisosteres, we conducted a structure-property relationship (SPR) study based on matched molecular pair (MMP) analyses. A series of representative examples have been characterized by experimentally determining physicochemical properties, such as acidity (p*K*_a_), lipophilicity (logD_7.4_), and permeability (PAMPA). The results presented can help estimate the relative changes in physicochemical properties that may be attainable by replacing the carboxylic acid functional group with fluorine containing surrogate structures.

The strategic deployment of fluorine atoms in biologically active molecules is an increasingly common tactic in drug design and optimization. The relatively small size of fluorine (*i.e.,*the Van Der Waals radius of fluorine is only approximately 20% larger than that of hydrogen), as well as its high electronegativity and ability to form C–F bonds that are more stable than the corresponding C–H or C–O bonds are often the primary characteristics underlying the success of fluorine in medicinal chemistry. In addition, the ability of fluorine to establish noncovalent interactions with the biological target *(e.g.,* H-bonds or multipolar interactions) and/or the possibility to impact conformational preferences as well as physicochemical properties *(e.g.,* lipophilicity and the p*K*_a_ of proximal functionalities) further underscore the multitude of opportunities presented by incorporation of fluorine in the context of drug design.^1^ Among the different applications of fluorine, one that continues to evolve is in the area of bioisosteric replacements.^2^ Fluorine has long been utilized as a possible replacement for hydrogen; moreover, the incorporation of one or multiple fluorine atoms in more elaborate structural motifs can be exploited in the design of surrogate structures of several functional groups. One such example is the use of fluorine in the construction of carboxylic acid bioisosteres. In this case, the impact of fluorine incorporation can have significant, but difficult to predict, effects on p*K*_a_, lipophilicity and permeability.

In this work, in order to compare the properties of fluorinated carboxylic acid surrogates with those of other commonly used nonfluorinated bioisosteres, we present the design and synthesis of a focused set of matched molecular pairs (MMPs), which include examples of substituted alcohols and phenols featuring one or more fluorine atoms, and the characterization of these compounds for key physicochemical properties such as acidity (p*K*_a_), lipophilicity (logD_7.4_ and permeability (logP*app*) in a parallel artificial membrane permeability assay (PAMPA). The data presented allow for an assessment of the impact of incorporation of fluorine on the physicochemical properties of carboxylic acid surrogates.

Previous studies from our labs investigated the structure-property relationship (SPR) of a collection of > 60 carboxylic acid and acylsulfonamide bioisosteres through the systematic evaluation of a set of MMPs based on the phenylpropionic acid template (**1**, Fig. 1A).^3-5^ Although relatively comprehensive, these studies included only a limited number of fluorine containing compounds, namely bis-orthofluorophenols, with no example of fluorinated aliphatic alcohols. Among the reasons for the limited number of fluorinated examples reported in our prior studies was that their relatively high lipophilicity (*i.e.,* logP/logD_7.4_ was often close to or above the upper limit of the measurable range), and poor aqueous solubility, hampered accurate property determinations. To circumvent this problem, in this study we evaluate model compounds derived from a substituted phenylpropionic acid featuring a polar group (*i.e.,* an amino group) in the *para* position (*i.e.,*., **2**, Fig. 1B) such that the lipophilicity of the fluorinated derivatives would remain within the measurable range while the increased polarity of the compounds would permit permeability determinations in the PAMPA assay.

A focused set of derivatives (**3**–**10**, Fig. 1B), including aliphatic alcohols exhibiting different degrees of fluorination (**3**–**6**), as well as phenols (**7**–**10**) was evaluated. The aliphatic fluorinated alcohols, **3**–**6**, were prepared using 4-nitrophenylpiOpionic acid **11** (Scheme 1) to incorporate the fluorinated functionality followed by reduction of the nitro group to the amine. The *gem*-di-CHF_2_ alcohol, **4**, was accessed in three steps through conversion of **11** to the corresponding acylchloride, **12**, followed by a nucleophilic difluoromethylation reaction^6^ to obtain **13**, followed by hydrogenation of the nitro group. The synthesis of **5** and **6** involved an initial reduction of **11** to the corresponding alcohol followed by oxidation to the aldehyde (**14**), which was then reacted with either the Ruppert-Prakash reagent, or difluoromethylene phosphoniumy lide^7^ to obtain alcohols **15** and **16**, respectively. Final hydrogenation of the nitro group of **15** and **16** afforded respectively **5** and **6**, with the latter compound producing crystals that were amenable to single-crystal X-ray diffraction (Scheme 1 and Supporting Information, CCDC number: 2246686). In a similar fashion, the synthesis of 3 was completed through Dess-Martin Periodinane (DMP)-mediated oxidation of **15** to the corresponding ketone (**17**), followed by trifluoromethylation with the Ruppert-Prakash reagent, and final hydrogenation reaction.

The synthesis of the fluorinated phenol derivatives **7**–**10** (Scheme 2) involved a S_N_Ar reaction of the appropriate fluoro-bromobenzene (**19**–**21**) with benzyl alcohol to give benzyl ethers **22**–**24**, followed by the introduction of a pinacolborane functionality (**25**–**27**). Next, SuzukiMiyaura reactions with the appropriate benzyl bromide followed by reduction of the nitro group led to model compounds **7**–**9** and **32** (Scheme 2). Further oxidation^8^ of the trifluoromethylsulfide **9** led to the corresponding sulfoxide **10**, however, all attempts to further oxidize **9** or **10** to the corresponding sulfone were not successful.

The data presented in Table 1 include the experimental values of logD_7.4_, p*K*_a_, and permeability as measured in a PAMPA assay. In addition, calculated values are also presented for comparison. As expected, incorporation of the amino group in **2** results in significant decrease in logD_7.4_ and permeability compared to the unsubstituted derivative **1**, giving us confidence that the reduced lipophilic character of the former compound would allow experimental measurements of the corresponding derivatives bearing fluorinated alcohols or phenols. The logD_7.4_ of fluorinated isosteres **3**–**10** was in the range of ~1–3 and, in the case of the fluorinated alcohols (**3**–**6**), the experimental values were in good agreement with the predicted values. However, in the case of fluorinated phenols, and especially for compounds **8**–**10**, the discrepancy between measured and calculated values was >2 log units, suggesting a caveat for using calculated values for these types of analogs. The lipophilicity determinations indicate a general trend linking the logD*7.4* values with the number of fluorine atoms, as exemplified by **3** and **6** being, respectively, the most and the least lipophilic examples within the set. However, the changes in lipophilicity did not always correlate with the number of fluorine atoms. For example, with a logD*7.4* of 1.83, the CF*3* derivative **5** is slightly more lipophilic than the derivative bearing a gem-diCHF*2* **4** (1.46). Similar observations have been previously reported^9^ and can be attributed to the formation of a larger dipole moment when the fluorine atoms are on vicinal carbons, as in *4*, that counteract the larger hydrophobic surface created by the presence of four fluorine atoms. With respect to the p*K*_a_, in general, all fluorinated derivatives exhibited weak acidic character with p*K*_a_ values ranging from about 8 to 12, with the polyfluorinated derivative **3** and sulfoxide phenol **10**, being the most acidic compounds (p*K* of 7.96 and 7.93 respectively). The difference between experimental and calculated values was relatively small (<1 log unit) apart from compounds **3** and **4** that showed a greater discrepancy of 1–1.7 log units. In agreement with the significant increase in lipophilicity and the limited acidic character, all fluorinated surrogates (alcohols or phenols), exhibit relatively high permeability values in the PAMPA assay in the range of 7 × 10^-6^ to 1 × 10^-5^ in contrast to the model compound **2**, which has low permeability (P_e_ < 1E-06 cm/s).

Finally, we evaluated whether the relative differences (Δ) in physicochemical properties introduced by each of the model compounds compared to the corresponding carboxylic acid control [*i.e.,* Δ = (property of isostere) – (property of corresponding –COOH)] could be used to compare the fluorinated derivatives to the previously described derivatives of the unsubstituted phenylpropionic acid. To do so, we used compound **32** as a matched pair analog of **7**: both bear the same carboxylic acid replacement (*i.e., ortho-*CF_3_ phenol) either on an aminosubstituted (7) or unsubstituted (32) phenylpropionic acid backbone. A comparison of the Δ p*K*_a_ and Δ logD_7.4_ values of **32** and **7**, shows that these values are within a relatively narrow range of ~0.5 log unit (*i.e.,* the difference in Δ p*K*_a_ and Δ logD_7.4_ values between the two compounds are respectively 0.46 and 0.52), suggesting that a comparison of the Δ values may be helpful to estimate the changes in physicochemical properties that could be expected with the different isosteric replacements.

As shown in Fig. 2 and in Supporting Information, when plotting the Δ values of model compounds **3**–**10** *vs* previously described phenylpropionic acid derivatives bearing carboxylic acid isosteres (**A**–**Y**), the fluorinated structures examined in this study impart a similarly weak acidic character as seen for other carboxylic acid isosteres, such as sulfonamide (**G1**, **G2**), hydroxamic ester (**B1**, **B2**), oxetan-3-ol (**Y**), thietane-3-ol (**X1**, **X2**), or acylurea (**J**) derivatives. However, with respect to the relative changes in lipophilicity and permeability, compared to the complete set of model compounds, the fluorinated derivatives impart the greatest increases as evidenced by Δ logD_7.4_ and Δ logP*app* values that are respectively > 3 and >1.5 log unit. Thus, taken together, these SPR data illustrate that the replacement of the carboxylic acid moiety with fluorinated alcohols or phenols is likely to result in significant lowering of the acidic character, and a concurrent increase lipophilicity and permeability. Depending on the particular context in which such replacements are utilized, this type of property changes may be desirable. For example, for carboxylic acid compounds that are meant to access the central nervous system (CNS) but exhibit limited permeability across the blood–brain barrier,^10^ a replacement with a fluorinated alcohol or phenol may result in more favorable brain exposure.

In summary, experimental determinations of acidity (p*K*_a_), lipophilicity (logD_7.4_), and PAMPA permeability have been conducted for a series of representative examples of fluorinated alcohols and phenols. The data generated in this study may help in characterizing the effect of fluorinations on the physicochemical properties of alcohols and phenols and in estimating the changes in physicochemical properties that may be attainable by replacing the carboxylic acid functional group with fluorine containing surrogate structures.

## Supplementary Material

Supporting Info1

Supporting Info2

## Figures and Tables

**Fig. 1.**
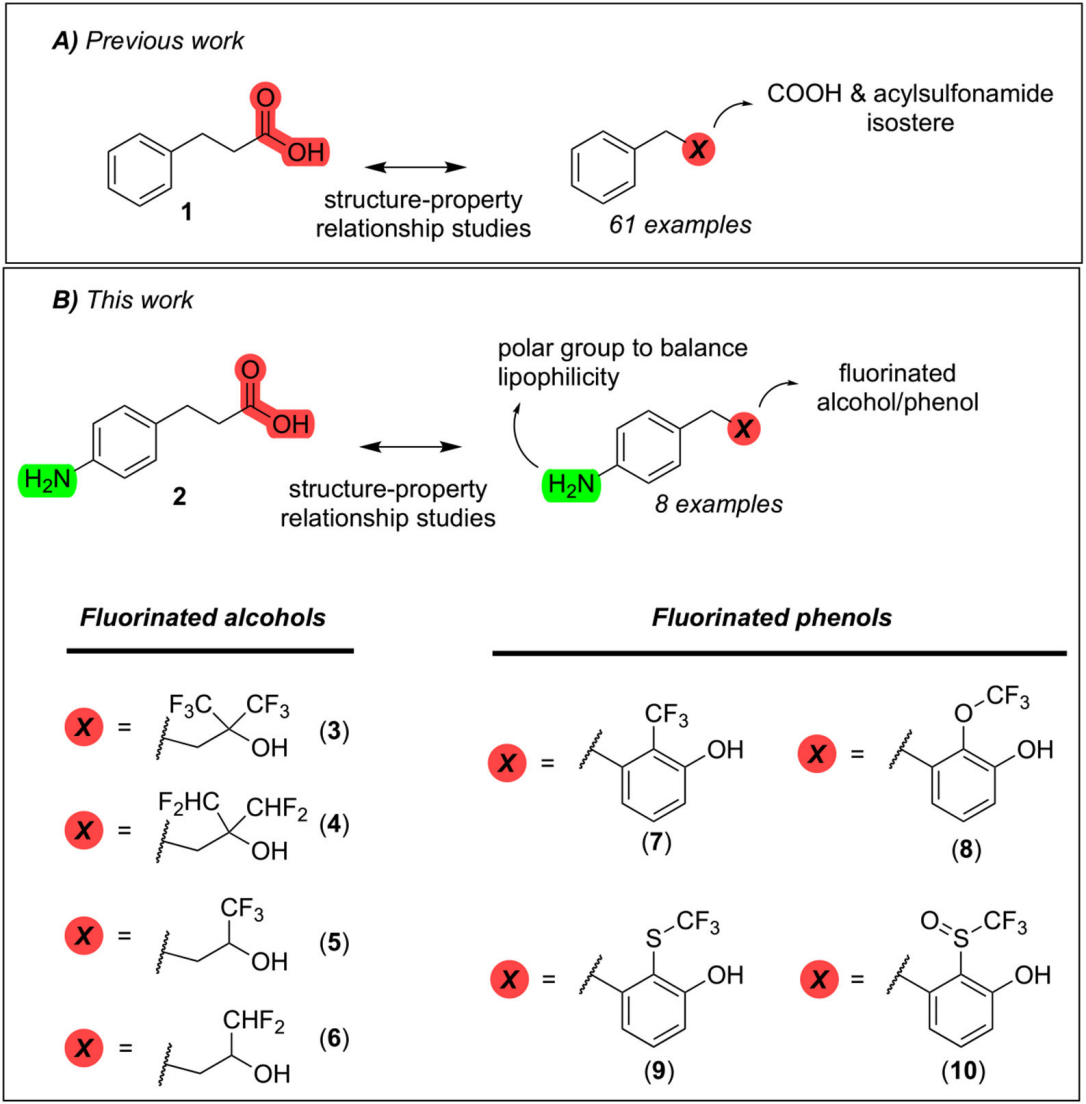
Schematic outline of previous work (*A*) and fluorinated model compounds evaluated for structure-property relationship studies (*B*).

**Fig. 2.**
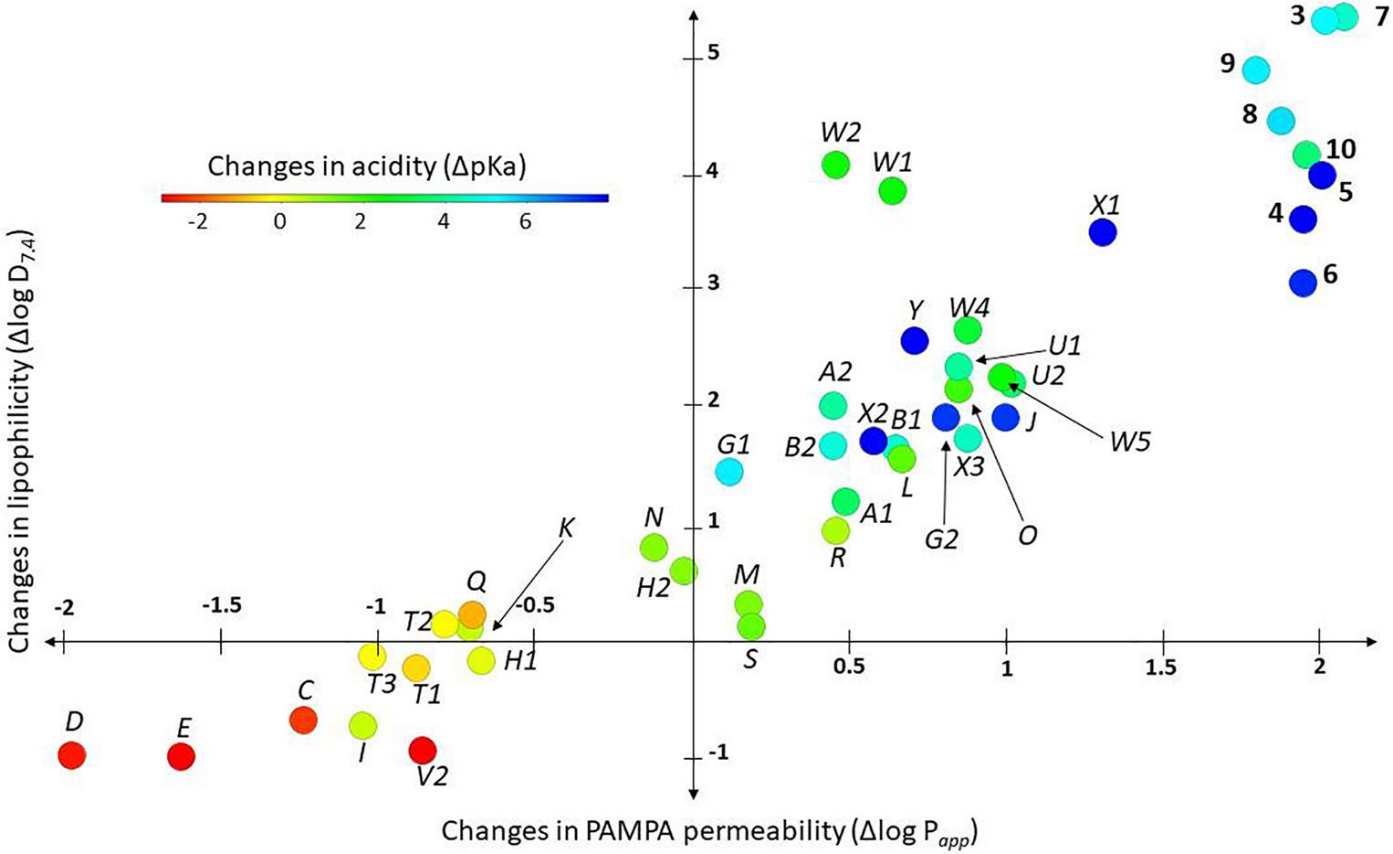
Plot showing the difference (Δ) in properties [*i.e.,* Δ = (property of isostere) – (property of corresponding –COOH)] of test compounds **3**–**10** and previously described phenylpropionic acid derivatives **A**–**Y** relative to the corresponding carboxylic acid. **A:** Hydroxamic acids, **B:** Hydroxamic esters, **C:** Phosphonic acid, **D:** Phosphinic acid, **E:** Sulfonic acid, **F:** Sulfinic acid, **G:** Sulfonamides, **H:** Acyl-sulfonamides, **I:** Sulfonylurea, **J:** Acylurea, **K:** Tetrazole, **L:** Thiazolidine dione, **M:** Oxazolidine dione, **N:** Oxadiazol-5(4*H*)-one, **O:** Thiadiazol-5(4*H*)-one, **P:** Oxathiadiazol-2-oxide, **Q:** Oxadiazol-5(4**H**)-thione, **R:** Isoxazole, **S:** Tetramic acid, **T:** Cyclopentane 1,3-diones, **U:** Cyclopentane 1,2-diones, **V:** Squaric acids, **W:** Substituted phenols, **X:** Thietane-3-ol derivatives, **Y:** Oxetan-3-ol. See Supporting Information for full data set and structures of model compounds **A**–**Y**. Numbers indicate cases were data for multiple examples of a specific bioisosteric replacement is available (*e.g.,* A, B, G, H, T, U, V, W, X).

**Scheme 1.**
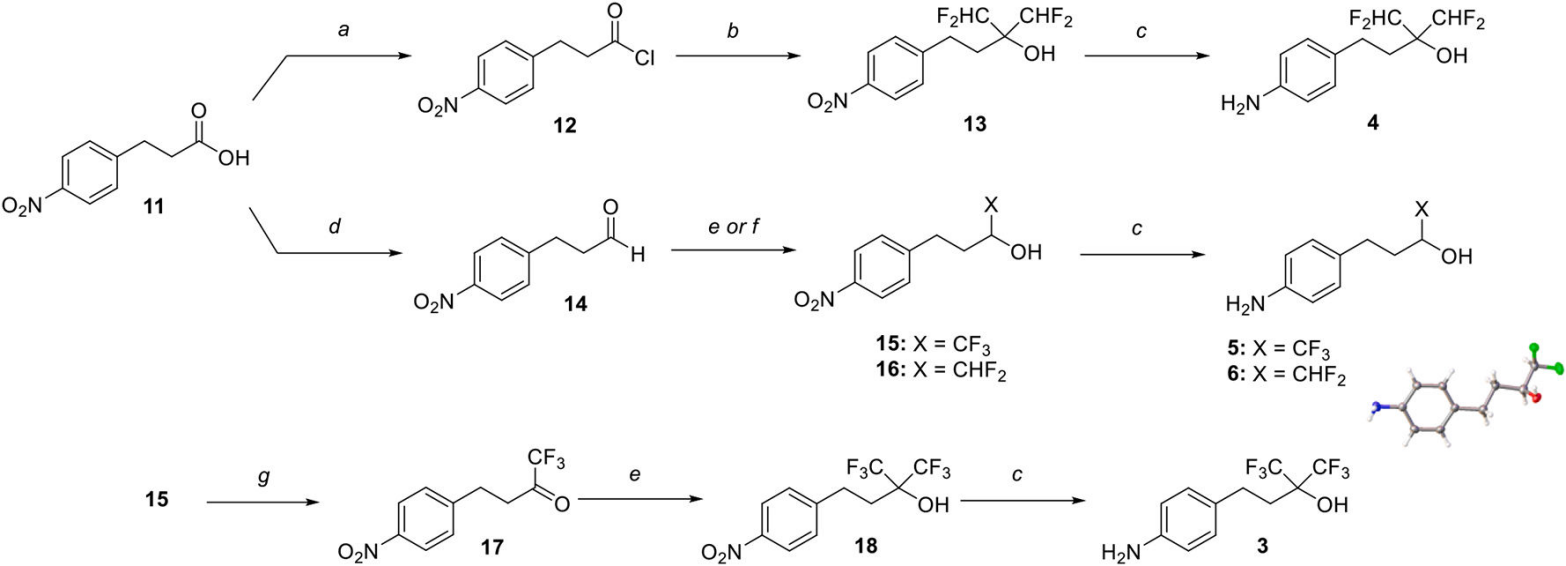
Reagents and Reaction Conditions: (a) oxalyl chloride, DMF, DCM, rt, 1 h, (99%); (b) i. (bromodifluoromethyl)trimethylsilane, triphenylphosphine, DMPU, MeCN, rt, 5 h; ii. Pyridine, H_2_O, 80 °C, 1.5 h, (44%); (c) H_2_ (1 atm), Pd/C, EtOAc, rt, 16 h, (**4:** 94%, **5:** 57%, **6:** 95%, **3:** 61%); (d) i. BH_3_, THF, 0 “C, 2 h, (99%); ii. DMP, DCM, rt, 16 h, (51%); (e) For 15 and **18:** CF_3_SiMe_3_, TBAF, THF, 0 °C to rt, 16 h, (**15:** 73%, **18:** 58%); (f) for **16:** Ph_3_P^+^CF_2_CO_2_, DMF, 50 °C, 1 h, (86%); (g) DMP, DCM, rt, 16 h, (81%).

**Scheme 2.**
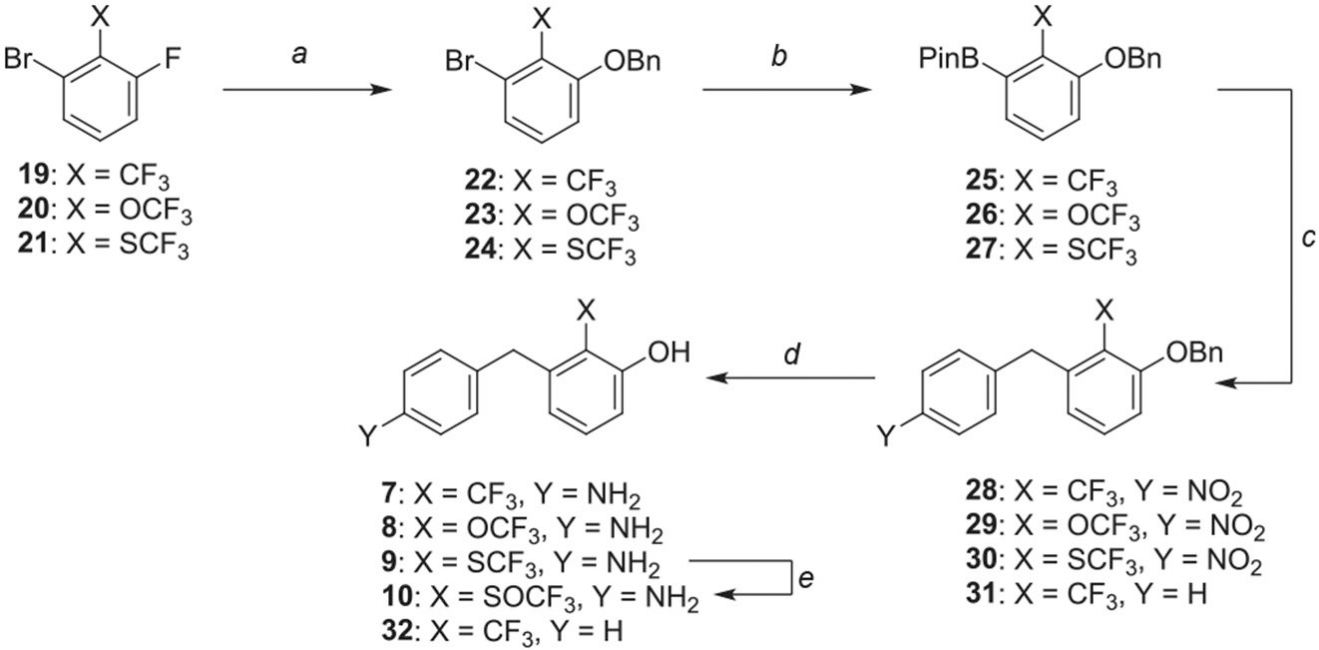
Reagents and Reaction Conditions: (a) BnOH, NaH, DMF, 0 °C to rt, 16 h (**22:** 91%, **23:** 98%, **24:** 87%); (b) B_2_Pin_2_, PdCl_2_dppf·DCM, AcOK, 1,4- dioxane, 80 °C, 3 h (**25:** 49%, **26:** 60%, **27:** 37%); (c) For **28**–**30**: 4-nitrobenzyl bromide, K_2_CO_3_, PdCl_2_dppf·DCM, DMF/H_2_O, 100 °C, 2 h, (**28:** 49%, **29:** 29%, **30:** 37%); For **31:** benzyl bromide, K_2_CO_3_, PdCl_2_dppf°DCM, DMF/H_2_O, 100 °C, 2 h (44%); (d) H_2_ (1 atm), Pd/C, MeOH, rt, 16 h (**7:** 81%, **8:** 94%, **9:** 57%, **32:** 98%); (e) H_2_O_2_, TFA, 60 °C, 3 h, (81%).

**Table 1:** Calculated and experimental properties of test compounds.

Cpd	Structure	logD_7.4_^a^ (calc.logD_7.4_)^b^	p*K*_a_^c^ (calc.p*K*_a_)^b^	PAMPA			
				Pe (cm/s)^d^	% retention^e^	logP *app*^f^	Permeability^g^
1	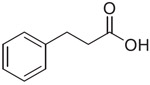	−0.49 (−0.56)	4.64 (4.73)	1.66E-06	−7	−5.79	+
2	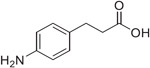	−2.13 (−1.29)	4.12 (3.61) *5.17(5.50)*	1.152E-07	0.05	−6.94	-
3	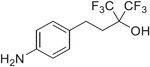	3.04 (2.82)	7.96 (8.98) *4.06 (4.08)*	1.202E-05	0.171	−4.92	+
4	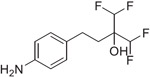	1.46 (1.39)	>12 (10.28) *4.79 (4.29)*	1.013E-05	0.02	−4.99	+
5	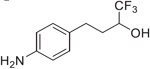	1.83 (2.12)	11.67 (11.70) *4.72 (4.31)*	1.166E-05	0.014	−4.93	+
6	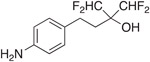	0.92 (1.40)	>12 (>12) *4.87 (4.47)*	1.025E-05	0.015	−4.99	+
7	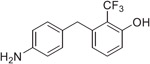	3.17 (3.91)	8.85 (8.51) *4.69 (3.99)*	1.377E-05	0.195	−4.86	+
8	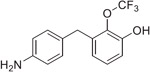	2.29 (4.74)	9.67 (9.72) *4.58 (3.92)*	8.6255E-06	0.36	−5.06	+
9	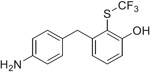	2.72 (5.12)	9.51 (8.82) *4.24 (3.98)*	7.2785E-06	0.51	5.14	+
10	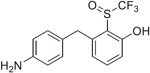	1.96 (4.31)	7.93 (8.02) *4.21 (3.90)*	1.0527E-05	0.24	−4.98	+
32	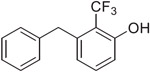	4.29 (4.61)	8.91 (8.51)	nd	nd	nd	nd

a Log of the distribution coefficient between *n*-octanol and aqueous buffer at pH 7.4 (logD_7.4_ determinations were conducted via potentiometric titrations using a Sirius T3, Pion, Inc.)

b Calculated values using ChemAxon

c p*K*_a_ values determined by potentiometric titrations using a Sirius T3, Pion, Inc. Values in italic correspond to the anilinium/aniline p*K*_a_.

d Effective permeability.

e Membrane retention.

f Log of the apparent permeability coefficient (P_app_) (PAMPA experiment ran by Analiza).

g Low permeability (—) is defined as a P_e_ value < 1.50E-6 cm/s, High permeability (+) is defined as a P_e_ value > 1.50E-6 cm/s. nd: not determined.

## Data Availability

Data will be made available on request.

## References

[1] Johnson BM, Shu YZ, Zhuo X, Meanwell NA. Metabolic and pharmaceutical aspects of fluorinated compounds. J Med Chem. 2020;63:6315–6386. 32182061 10.1021/acs.jmedchem.9b01877

[2] Meanwell NA. Fluorine and fluorinated motifs in the design and application of bioisosteres for drug design. J Med Chem. 2018;61:5822–5880. 29400967 10.1021/acs.jmedchem.7b01788

[3] Francisco KR, Varricchio C, Paniak TJ, Kozlowski MC, Brancale A, Ballatore C. Structure property relationships of N-acylsulfonamides and related bioisosteres. Eur J Med Chem. 2021;218, 113399. 33823393 10.1016/j.ejmech.2021.113399 PMC8105289

[4] Lassalas P, Oukoloff K, Makani V, Evaluation of oxetan-3-ol, thietan-3-ol, and derivatives thereof as bioisosteres of the carboxylic acid functional group. ACS Med Chem Lett. 2017;8:864–868. 28835803 10.1021/acsmedchemlett.7b00212 PMC5554911

[5] Lassalas P, Gay B, Lasfargeas C, Structure property relationships of carboxylic acid isosteres. J Med Chem. 2016;59:3183–3203. 26967507 10.1021/acs.jmedchem.5b01963 PMC4833640

[6] Trifonov AL, Zemtsov AA, Levin VV, Struchkova MI, Dilman AD. Nucleophilic difluoromethylation using (bromodifluoromethyl)trimethylsilane. Org Lett. 2016;18: 3458–3461. 27336618 10.1021/acs.orglett.6b01641

[7] Levin VV, Trifonov AL, Zemtsov AA, Struchkova MI, Arkhipov DE, Dilman AD. Difluoromethylene phosphabetaine as an equivalent of difluoromethyl carbanion. Org Lett. 2014;16:6256–6259. 25423177 10.1021/ol503225s

[8] Horvat M, Kodric G, Jereb M, Iskra J. One pot synthesis of trifluoromethyl aryl sulfoxides by trifluoromethylthiolation of arenes and subsequent oxidation with hydrogen peroxide. RSC Adv. 2020;10:34534–34540. 35514387 10.1039/d0ra04621c PMC9056837

[9] Jeffries B, Wang Z, Felstead HR, Systematic investigation of lipophilicity modulation by aliphatic fluorination motifs. J Med Chem. 2020;63:1002–1031. 31894985 10.1021/acs.jmedchem.9b01172

[10] Pajouhesh H, Lenz GR. Medicinal chemical properties of successful central nervous system drugs. NeuroRx. 2005;2:541–553. 16489364 10.1602/neurorx.2.4.541 PMC1201314

