# Supplementary material for: Structure-property relationships of fluorinated carboxylic acid bioisosteres
Source: Bioorg Med Chem Lett. Author manuscript; Available in PMC 2024 Apr 9. (PMC11003450; doi:10.1016/j.bmcl.2023.129363)

Structure property relationships of fluorinated carboxylic acid bioisosteres.

Thibault Alle,[a] Sipak Joyasawal,[b] Killian Oukoloff,[a] Keith Long,[b] Zachary Owyang,[a] Karol R. Francisco,[a] Dominique Cahard,[c,*] Donna M. Huryn,[b,*] Carlo Ballatore[a,*]

[a] *Skaggs School of Pharmacy and Pharmaceutical Sciences, University of California, San Diego, 9500 Gilman Drive, La Jolla, CA 92093*
[b] *Department of Pharmaceutical Sciences, University of Pittsburgh School of Pharmacy, 3501 Terrace St., Pittsburgh, PA 15261*
[c] *CNRS, UMR 6014 COBRA, Normandie Université, 76821 Mont Saint Aignan, France*

Supporting Information

NMR description of final compounds……………………………………………………….Page 2-3
X-Ray report of compound **6**……...……………….……………………………...……Page 4-7
pKa and LogD Sirius data…………………………….…..……………………….Page 8-27

4-(4-Aminophenyl)-1,1,1-trifluoro-2-(trifluoromethyl)butan-2-ol (3) ^{1}H NMR (400 MHz, $CDCl_3$): δ 7.00 (d, 2H), 6.64 (d, 2H), 3.88 (bs, 1H) 3.59 (bs, 2H) 2.84 – 2.77 (m, 1H), 2.68 – 2.63 (m, 1H), 1.98 – 1.89 (m, 3H) ppm. ^{13}C NMR (100 MHz, $CDCl_3$): δ 144.4, 130.2, 129.61, 123.2 (q, *J* = 288 Hz), 115.8, 76.2 (q, *J* = 29 Hz), 32.3, 27.4 ppm; MS(ESI) m/z for $C_{11}H_{11}F_6NO$ (287.0) observed $[M+H]^+$: 288.4.

4-(4-Aminophenyl)-2-(difluoromethyl)-1,1-difluorobutan-2-ol (4) ^{1}H NMR (300 MHz, $CDCl_3$) δ 7.00 (d, *J* = 8.1 Hz, 1H), 6.64 (d, *J* = 8.3 Hz, 1H), 6.05 – 5.62 (m, 1H), 2.92 – 2.49 (m, 1H), 2.23 – 1.90 (m, 1H) ppm. MS(ESI) m/z for $C_{11}H_{13}F_4NO$ (251.1) observed $[M+H]^+$: 252.2.

4-(4-Aminophenyl)-1,1,1-trifluorobutan-2-ol (5) ^{1}H NMR (400 MHz, $CDCl_3$): δ 7.00 (d, *J* = 7Hz, 2H), 6.64 (d, *J* = 6.7 Hz, 2H), 3.88 (bs, 1H) 3.59 (bs, 2H) 2.84-2.77 (m, 1H), 2.68-2.63 (m, 1H), 1.98 – 1.89 (m, 3H) ppm. ^{13}C NMR (100 MHz, $CDCl_3$): δ 145.0, 130.6, 129.6, 125.6 (q, *J* = 280 Hz), 115.8, 69.9 (q, *J* = 31 Hz), 31.6, 30.3 ppm; MS(ESI) m/z for $C_{10}H_{12}F_3NO$ (219.0) observed $[M+H]^+$: 220.2.

4-(4-Aminophenyl)-1,1-difluorobutan-2-ol (6) ^{1}H NMR (300 MHz, $CDCl_3$) : δ = 7.01 (d, *J* = 8.1 Hz, 2H), 6.65 (d, *J* = 8.1 Hz, 2H), 5.61 (td, *J* = 56.1, 4.2 Hz, 1H), 3.81 – 3.64 (m, 1H), 2.86 – 2.71 (m, 1H), 2.71 – 2.56 (m, 1H), 1.86 – 1.69 (m, 2H) ppm. ^{13}C NMR (75.5 MHz, $CDCl_3$) : δ = 144.4, 130.9, 129.2, 116.4 (t, *J* = 243.7 Hz), 115.4, 70.2 (t, *J* = 23.1 Hz), 31.7 (t, *J* = 2.8 Hz), 30.0 ppm. HRMS (ESI): Calculated for $C_{12}H_{17}N_2OF_2$ $[M+H+CH_3CN]^+$: 243.1309; observed: 243.1318.

3-(4-Aminobenzyl)-2-(trifluoromethyl)phenol (7) ^{1}H NMR (600 MHz, $CDCl_3$) δ 7.06 – 6.99 (m, 1H), 6.90 (d, *J* = 8.0 Hz, 2H), 6.83 (d, *J* = 8.7 Hz, 1H), 6.73 (d, *J* = 7.6 Hz, 1H), 6.64 (d, *J* = 8.0 Hz, 2H), 4.03 (s, 2H) ppm. ^{13}C NMR (151 MHz, CD_3CN) δ 157.10, 147.00, 142.74, 133.43, 130.11, 130.00, 127.16, 125.34, 124.67, 116.09, 115.45, 39.21 ppm. HRMS (ES+) calculated for $C_{14}H_{13}F_3NO_2S$ $[M + H]^+$ 316.0614, found 316.0617.

3-(4-Aminobenzyl)-2-(trifluoromethoxy)phenol (8) ^{1}H NMR (600 MHz, $CDCl_3$) δ 7.07 (t, *J* = 7.9 Hz, 1H), 6.95 (d, *J* = 8.3 Hz, 2H), 6.86 (dd, *J* = 8.1, 1.6 Hz, 1H), 6.66 (dd, *J* = 7.7, 1.6 Hz, 1H), 6.63 (d, *J* = 8.3 Hz, 2H), 3.90 (s, 2H), 3.59 (bs, 2H) ppm. ^{13}C NMR (151 MHz, CD_3CN) δ 150.81, 147.23, 138.43, 130.45, 129.30, 128.81, 122.62, 116.03, 115.50, 35.27 ppm.

HRMS (ES+) calculated for $C_{14}H_{13}F_3NO_2S$ $[M + H]^+$ 316.0614, found 316.0617.

3-(4-Aminobenzyl)-2-((trifluoromethyl)thio)phenol (9) ^{1}H NMR (600 MHz, $CDCl_3$) δ 7.32 (t, *J* = 7.9 Hz, 1H), 6.94 (d, *J* = 8.0 Hz, 3H), 6.79 (d, *J* = 7.6 Hz, 1H), 6.62 (d, *J* = 7.4 Hz, 2H), 4.17 (s, 2H) ppm. ^{13}C NMR (151 MHz, $CDCl_3$) δ 158.83, 148.91, 133.62, 130.00, 127.92, 122.90, 115.55 (dt, *J* = 5.2, 2.6 Hz), 113.93, 108.54, 39.52 ppm. HRMS (ES+) calculated for $C_{14}H_{13}F_3NOS$ $[M + H]^+$ 300.0664, found 300.065.

3-(4-Aminobenzyl)-2-((trifluoromethyl)sulfinyl)phenol (10) ^{1}H NMR (600 MHz, $CDCl_3$) δ 7.38 (t, *J* = 8.0 Hz, 1H), 6.89 (d, *J* = 8.0 Hz, 2H), 6.85 (d, *J* = 8.4 Hz, 1H), 6.71 (d, *J* = 7.5 Hz, 1H), 6.64 (d, *J* = 8.5 Hz, 2H), 3.97 (d, *J* = 16.1 Hz, 1H), 3.81 (d, *J* = 16.1 Hz, 1H) ppm. ^{13}C NMR (151 MHz, $CDCl_3$) δ 163.05, 145.36, 142.92, 135.73, 129.88, 127.97, 125.94 (q, *J* = 338.2 Hz), 122.58, 118.52, 115.60, 112.41, 37.22 ppm. HRMS (ES+) calculated for $C_{14}H_{13}F_3NO_2S$ $[M + H]^+$ 316.0614, found 316.0617.

3-Benzyl-2-(trifluoromethyl)phenol (32) ^{1}H NMR (600 MHz, $CDCl_3$) δ 7.32 – 7.27 (m, 3H), 7.21 (t, *J* = 7.4 Hz, 1H), 7.10 (d, *J* = 7.5 Hz, 2H), 6.86 (d, *J* = 8.3 Hz, 1H), 6.74 (d, *J* = 7.6 Hz, 1H), 5.95 (q, *J* = 6.5 Hz, 1H), 4.15 (s, 2H) ppm. ^{13}C NMR (151 MHz, $CDCl_3$) δ 154.72, 141.02, 140.13, 132.81, 128.90, 128.59, 126.38, 124.71, 116.82, 114.43 (d, *J* = 27.6 Hz), 39.13 ppm.

X-ray report - Experimental Summary

The single crystal X-ray diffraction studies were carried out on a Bruker Kappa APEX-II CCD diffractometer equipped with Mo K_α radiation (λ = 0.71073). Crystals of the subject compound were grown by dissolving approximately 5mg of sample in 1mL of Dichloromethane, which was then vapor diffused with Pentane over one week. A 0.150 x 0.075 x 0.060 mm piece of a colorless block was mounted on a Cryoloop with Paratone oil. Data were collected in a nitrogen gas stream at 100.0 K using ϕ and ϖ scans. Crystal-to-detector distance was 40 mm using variable exposure time (2s-5s) depending on θ with a scan width of 1.0°. Data collection was 100% complete to 57° in θ. A total of 10170 reflections were collected covering the indices, $-11 \leq h \leq 12$, $-7 \leq k \leq 7$, $-18 \leq l \leq 18$. 1810 reflections were found to be symmetry independent, with a R_{int} of 0. 0287. Indexing and unit cell refinement indicated a primitive, monoclinic lattice. The space group was found to be $P2_1/c$. The data were integrated using the Bruker SAINT software program and scaled using the SADABS software program. Solution by direct methods (SHELXL) produced a complete phasing model consistent with the proposed structure.

Compound 6 (CCDC number : 2246686)

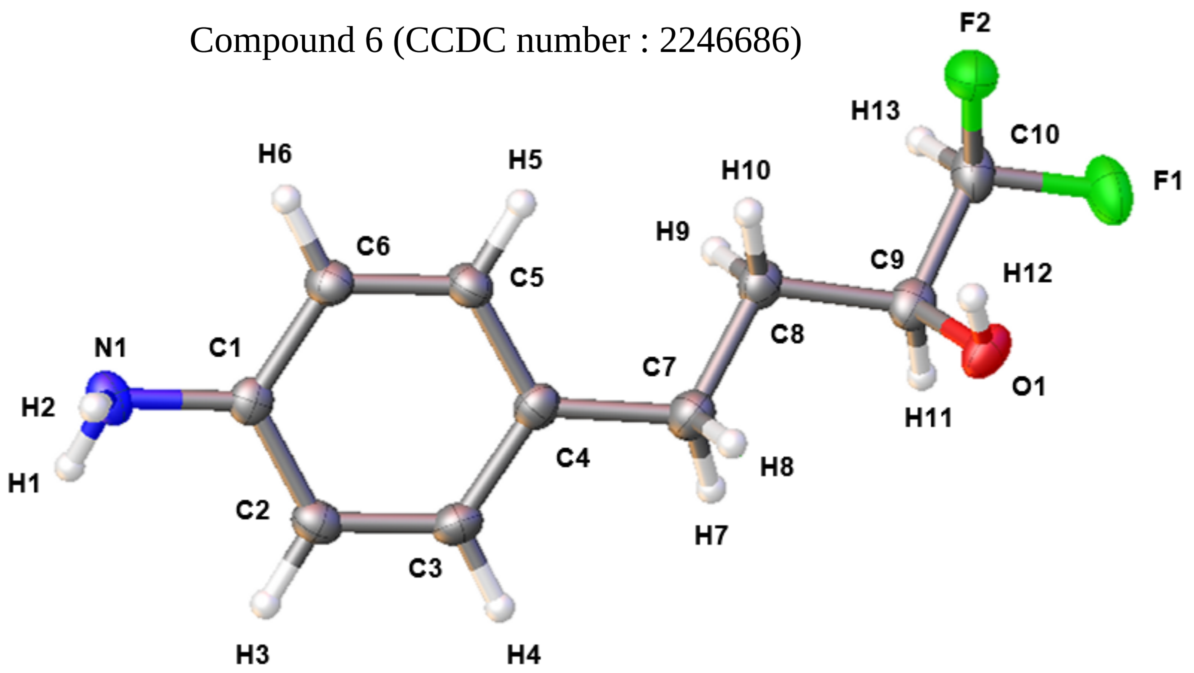

Table 1 Crystal data and structure refinement for BL_0583 (compound 6)

Identification code	BL_0583
Empirical formula	$C_{10}H_{13}F_2NO$
Formula weight	201.21
Temperature/K	100.0
Crystal system	monoclinic
Space group	$P2_1/c$
a/Å	10.1090(6)
b/Å	6.4519(4)
c/Å	15.4301(10)
α/°	90
β/°	101.631(2)
γ/°	90
Volume/Å^3	985.72(11)
Z	22
ρ_{calc}g/cm^3	7.457
μ/mm^{-1}	0.621
F(000)	2332.0
Crystal size/mm^3	0.15 × 0.075 × 0.06
Radiation	MoKα (λ = 0.71073)
2Θ range for data collection/°	4.114 to 50.736
Index ranges	$-11 \leq h \leq 12$, $-7 \leq k \leq 7$, $-18 \leq l \leq 18$
Reflections collected	10170
Independent reflections	1810 [R_{int} = 0.0287, R_{sigma} = 0.0204]
Data/restraints/parameters	1810/0/129
Goodness-of-fit on F^2	1.447
Final R indexes [I>=2σ (I)]	R_1 = 0.0485, wR_2 = 0.1690
Final R indexes [all data]	R_1 = 0.0567, wR_2 = 0.1792
Largest diff. peak/hole / e Å^{-3}	0.79/-0.68

Table 2 Fractional Atomic Coordinates (×10^4) and Equivalent Isotropic Displacement Parameters (Å^2×10^3) for BL_0583. U_{eq} is defined as 1/3 of the trace of the orthogonalised U_{IJ} tensor.

Atom	*x*	*y*	*z*	U(eq)
F1	9650.5(12)	7288(2)	5374.5(9)	38.1(4)
F2	8408.2(11)	8367.4(17)	6287.3(8)	32.7(4)
O1	9195.4(12)	4259(2)	6575.4(9)	27.7(4)
N1	1058.2(15)	739(3)	6750.0(10)	23.6(4)
C1	2354.8(18)	1176(3)	6599.2(12)	20.0(5)
C2	3124.7(19)	-342(3)	6293.5(13)	23.2(5)
C3	4414(2)	104(3)	6168.6(13)	24.1(5)
C4	4988.0(18)	2058(3)	6344.3(12)	22.3(5)
C5	4203.7(18)	3572(3)	6653.1(12)	23.1(5)
C6	2912.7(19)	3143(3)	6776.2(12)	22.4(5)
C7	6427(2)	2442(3)	6237.8(14)	27.5(5)
C8	6752.6(19)	4657(3)	6019.9(13)	23.3(5)
C9	8175.2(19)	4878(3)	5849.9(13)	25.0(5)
C10	8403.2(19)	7071(3)	5583.5(14)	27.6(5)

Table 3 Anisotropic Displacement Parameters (Å^2×10^3) for BL_0583. The Anisotropic displacement factor exponent takes the form: $-2\pi^2[h^2a^{*2}U_{11}+2hka^*b^*U_{12}+\ldots]$.

Atom	U_{11}	U_{22}	U_{33}	U_{23}	U_{13}	U_{12}
F1	33.3(8)	47.4(8)	38.1(8)	-8.9(6)	17.7(6)	-14.7(5)
F2	31.5(7)	27.4(7)	40.6(8)	-5.7(5)	10.7(6)	-1.7(5)
O1	20.2(8)	35.3(8)	25.8(8)	-4.2(6)	0.8(6)	6.8(6)
N1	24.2(9)	28.6(9)	17.2(9)	-1.3(7)	2.2(7)	-6.5(7)
C1	19.9(10)	24.5(10)	14.7(9)	1.1(7)	1.2(7)	0.8(7)
C2	26.9(11)	20.2(10)	21.3(10)	-0.4(7)	1.8(8)	-1.4(7)
C3	25.8(11)	20.9(10)	25.3(11)	-0.4(8)	4.9(8)	5.0(7)
C4	21.5(10)	23.5(10)	21.6(10)	2.1(8)	3.5(8)	3.2(7)
C5	25.2(11)	20.2(10)	24.4(11)	-2.1(8)	6.1(8)	-2.6(7)
C6	25.7(10)	22.6(10)	19.6(10)	-2.5(8)	6.4(8)	0.2(8)
C7	24.9(11)	27.1(11)	31.4(12)	0.9(9)	7.6(9)	2.4(8)
C8	19.7(10)	26.3(10)	22.7(11)	-0.7(8)	1.7(8)	1.0(7)
C9	22.6(11)	30.9(11)	20.5(11)	-5.6(8)	1.8(8)	-0.2(8)
C10	21.6(11)	37.0(11)	24.5(11)	-3.0(9)	5.4(8)	-4.6(8)

Table 4 Bond Lengths for BL_0583.

Atom	Atom	Length/Å	Atom	Atom	Length/Å

F1	C10	1.370(2)	C1	C6	1.393(3)
F2	C10	1.370(2)	C1	C2	1.391(3)
O1	C9	1.418(2)	C2	C3	1.385(3)
N1	C1	1.405(2)	C5	C6	1.384(3)
C4	C5	1.401(3)	C8	C7	1.519(3)
C4	C3	1.392(3)	C8	C9	1.520(3)
C4	C7	1.517(3)	C9	C10	1.504(3)

Table 5 Bond Angles for BL_0583.

Atom	Atom	Atom	Angle/°	Atom	Atom	Atom	Angle/°
C5	C4	C7	122.96(17)	C2	C3	C4	121.87(18)
C3	C4	C5	117.10(18)	C7	C8	C9	112.39(16)
C3	C4	C7	119.88(17)	C4	C7	C8	115.63(16)
C2	C1	N1	121.27(17)	O1	C9	C8	113.60(16)
C2	C1	C6	118.40(17)	O1	C9	C10	110.71(16)
C6	C1	N1	120.31(16)	C10	C9	C8	109.74(15)
C3	C2	C1	120.49(17)	F2	C10	F1	105.54(14)
C6	C5	C4	121.41(17)	F2	C10	C9	109.51(17)
C5	C6	C1	120.73(17)	F1	C10	C9	111.06(16)

Table 6 Hydrogen Atom Coordinates (Å×10^4) and Isotropic Displacement Parameters (Å^2×10^3) for BL_0583

Atom	*x*	*y*	*z*	U(eq)
H1	715.21	-330.04	6420.85	28
H2	1105.22	425.56	7313.63	28
H3	2763.85	-1694	6169.53	28
H4	4919.92	-954.94	5956.9	29
H5	4565.52	4922.46	6780.71	28
H6	2401.47	4201.16	6983.67	27
H7	6615.98	1525.56	5762.48	33
H8	7046.25	2032.68	6793.43	33
H9	6097.25	5118.85	5488.9	28
H10	6651.42	5570.79	6517.91	28
H11	8245.16	3961.91	5338.24	30
H12	9101.44	4907.77	7031.06	41
H13	7681.22	7494.69	5071.01	33

pH-metric Result

Sample name:	**4-Aminophenylpropionic acid**	Authentication:	**Failed**
Assay name:	**pH-metric pKa**	Experiment start time:	**11/9/2017 12:22:23 PM**
Assay ID:	**17K-09002**	Analyst:	**CB**
Quality:	**Good**	Instrument ID:	**T317135**
Filename:	**C:\Users\labuser\Desktop\Fluorinated isosteres\4-aminopropionic acid pKa.t3r**		

pH-metric Result

Acid pKa 1	4.12	±0.00 (n=50)
Base pKa 2	5.17	±0.01 (n=50)
RMSD	0.055	

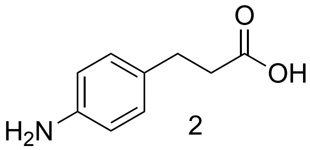
2

Warnings and errors

Errors	None
Warnings	None

Graphs

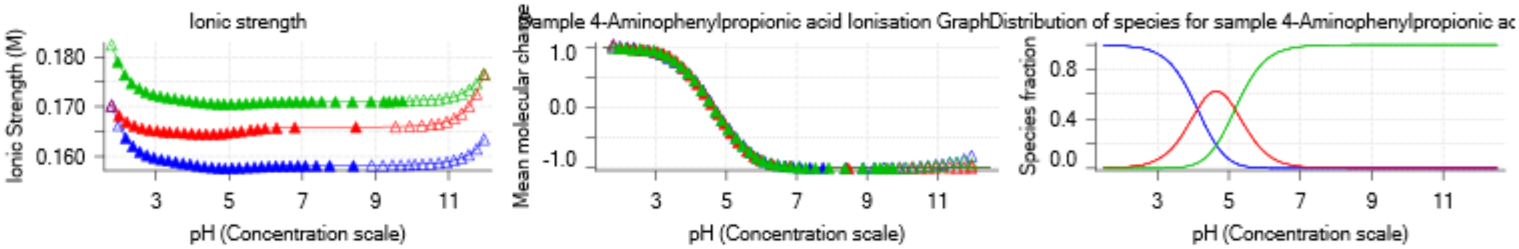

pH-metric Result

Sample name:	**4-Aminophenylpropionic Acid logP**	Experiment start time:	**11/14/2017 4:55:37 PM**
Assay name:	**pH-metric high logP**	Analyst:	**9.000**
Assay ID:	**17K-14003**	Instrument ID:	**T317135**
Filename:	**C:\Users\labuser\Desktop\Fluorinated isosteres\4-aminopropionic acid logP.t3r**		

pH-metric Result

logP (XH2 +)	-1.10	±1.24 (n=50)
logP (neutral XH)	0.10	±0.02 (n=50)
logP (X -)	-10.00	
RMSD	0.202	

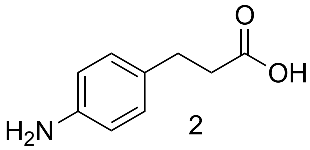

2

Warnings and errors

Errors	None
Warnings	None

Sample logD values

pH	4-Aminophenylpropionic Acid logP logD	Comment
1.000	-1.10	
1.200	-1.10	Stomach pH
2.000	-1.06	
3.000	-0.79	
4.000	-0.25	
5.000	-0.16	
6.000	-0.79	
6.500	-1.25	
7.000	-1.74	
7.400	-2.13	Blood pH
8.000	-2.73	
9.000	-3.73	
10.000	-4.73	
11.000	-5.73	
12.000	-6.73	

Graphs

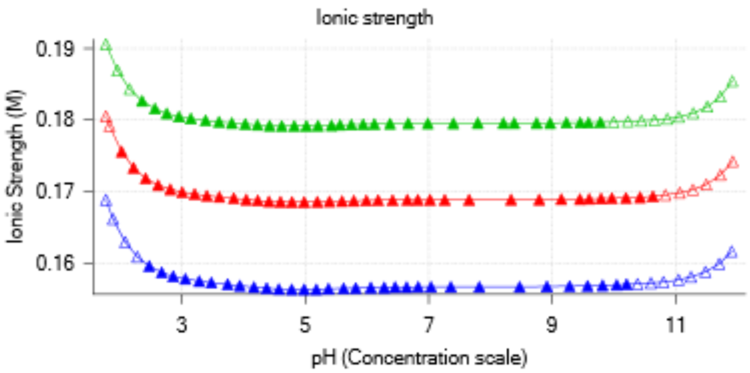

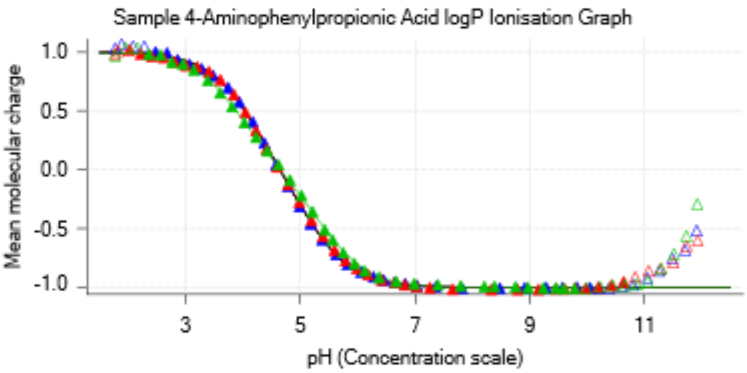

Yasuda-Shedlovsky Result

Sample name:	**BL-0618_2**	Experiment start time:	**5/4/2018 3:55:27 PM**
Assay name:	**pH-metric pKa**	Analyst:	
Assay ID:	**18E-04005**	Instrument ID:	**T317135**
Filename:	**C:\Sirius Data Backup\18E-04005_BL-0618_2_pH-metric pKa.t3r**		

Yasuda-Shedlovsky result

Extrapolation type	pKa 0%	SD	Intercept	Slope	R^2	Ionic strength	Temperature
Yasuda-Shedlovsky	4.06	±0.00	6.66	-67.3965	0.9999	0.170 M	25.0°C
Yasuda-Shedlovsky	7.96	±0.00	10.56	-67.3965	0.9999	0.170 M	25.0°C

Warnings and errors

Errors None
Warnings None

Graphs

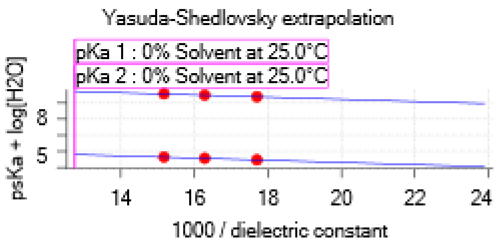

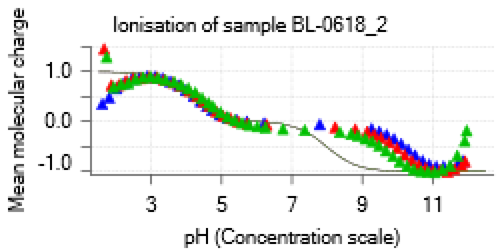

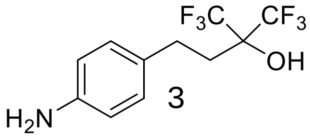

3

pH-metric Result

RMSD 1.414

Warnings and errors

Errors None
Warnings None

Graphs

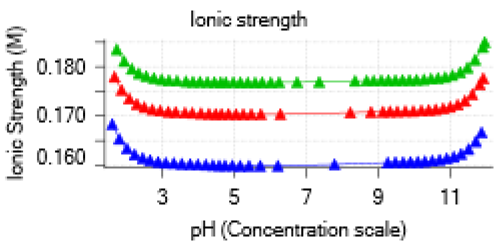

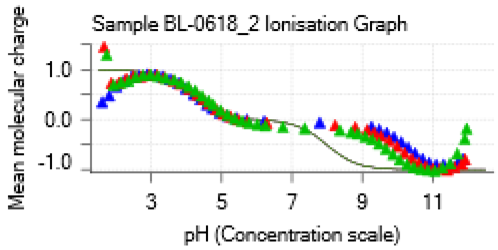

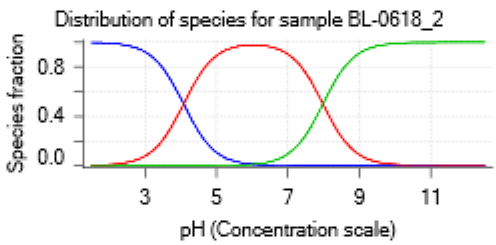

pH-metric Result

Multiset name:
Filename:

Instrument ID: **T317135**

pH-metric Result

logP (XH2 +)	-10.00
logP (neutral XH)	3.05
logP (X -)	-10.00
RMSD	31.697

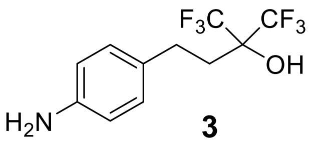

3

Warnings and errors

Errors	None
Warnings	None

Sample logD values

pH	BL-0618 logD	Comment
1.000	-0.83	
1.200	-0.63	Stomach pH
2.000	0.17	
3.000	1.16	
4.000	2.12	
5.000	2.80	
6.000	3.02	
6.500	3.04	
7.000	3.04	
7.400	3.04	Blood pH
8.000	3.03	
9.000	2.90	
10.000	2.34	
11.000	1.42	
12.000	0.43	

Graphs

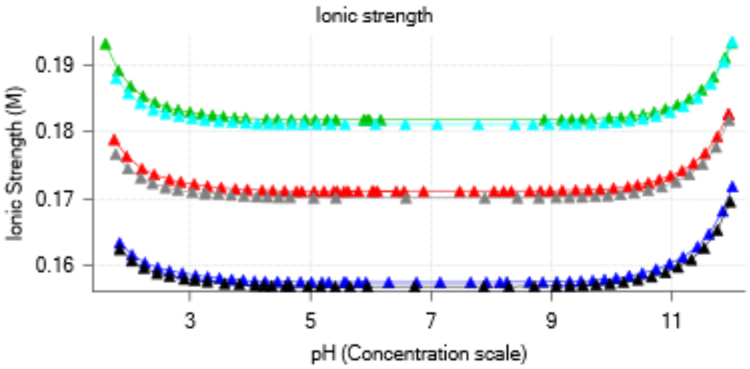

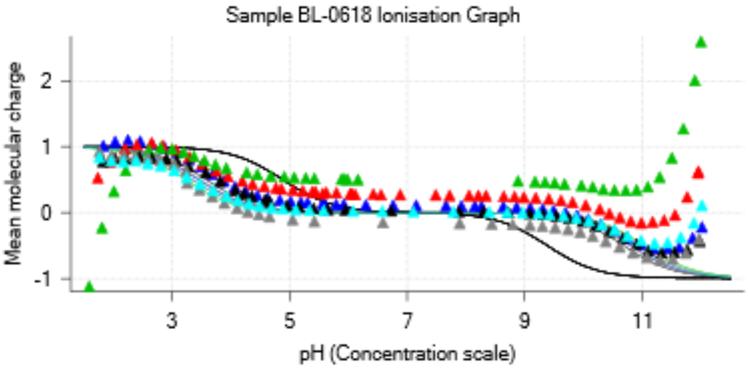

pH-metric Result

Sample name: **BL-0727**
Assay name: **pH-metric pKa**
Assay ID: **19F-17003**
Filename:

Experiment start time: **6/17/2019 4:07:05 PM**
Analyst:
Instrument ID: **T317135**

pH-metric Result

Base pKa 1 4.79
RMSD 0.178

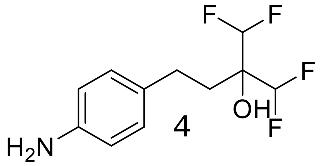

4

Warnings and errors

Errors None
Warnings None

Graphs

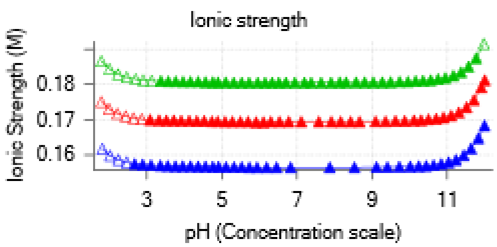

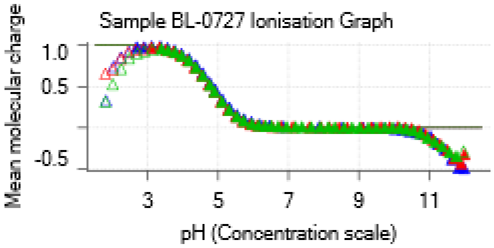

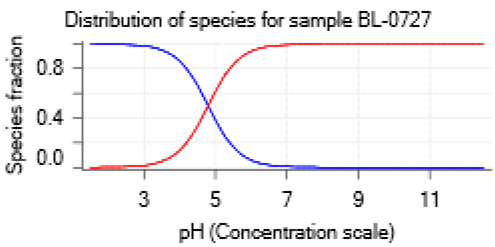

Sample name:	**BL-0727**	Experiment start time:	**6/18/2019 5:20:07 PM**
Assay name:	**pH-metric medium logP**	Analyst:	
Assay ID:	**19F-18005**	Instrument ID:	**T317135**
Filename:			

pH-metric Result

logP (XH2 +)	-0.44
logP (neutral XH)	1.46
logP (X -)	-10.00
RMSD	0.395

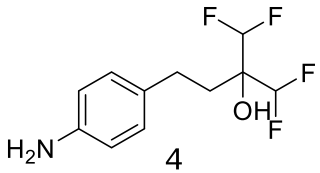

4

Warnings and errors

Errors	None
Warnings	None

Sample logD values

**pH**	**BL-0727 logD**	**Comment**
1.000	-0.44	
1.200	-0.43	Stomach pH
2.000	-0.39	
3.000	-0.09	
4.000	0.64	
5.000	1.25	
6.000	1.43	
6.500	1.45	
7.000	1.46	
7.400	1.46	Blood pH
8.000	1.46	
9.000	1.46	
10.000	1.45	
11.000	1.41	
12.000	1.15	

Graphs

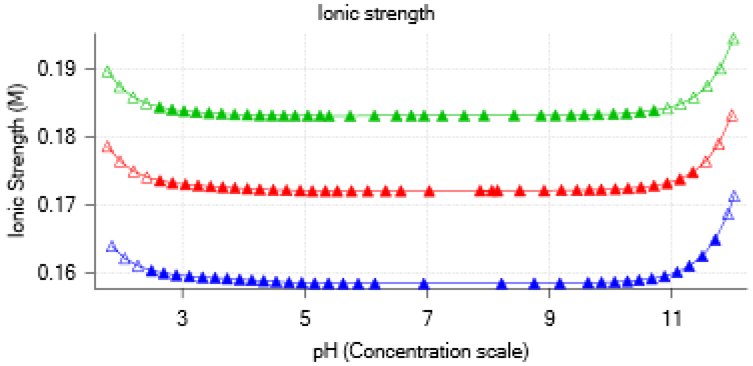

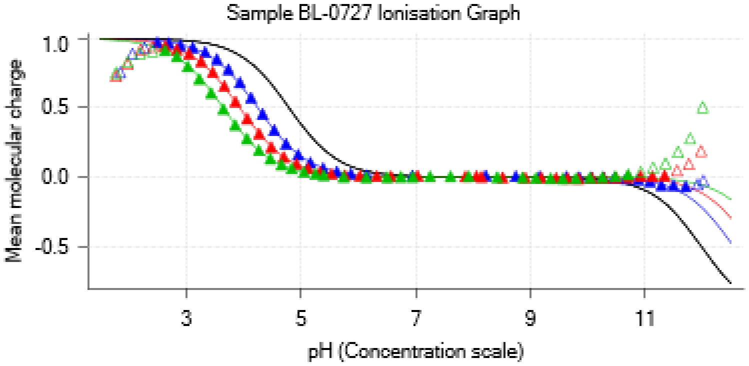

Yasuda-Shedlovsky Result

Multiset name:
Filename:

Instrument ID: **T317135**

Yasuda-Shedlovsky result

Extrapolation type	pKa 0%	SD	Intercept	Slope	R^2	Ionic strength	Temperature
Yasuda-Shedlovsky	4.72	±0.35	8.09	-127.6466	0.2478	0.171 M	25.0°C
Yasuda-Shedlovsky	11.67	±0.24	14.78	-107.7450	0.3414	0.171 M	25.0°C

Warnings and errors

Errors None
Warnings None

Graphs

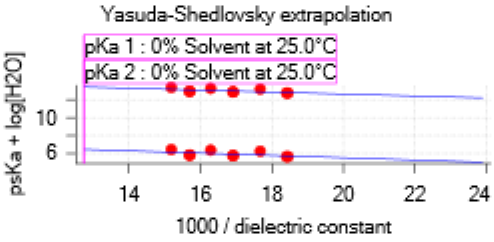

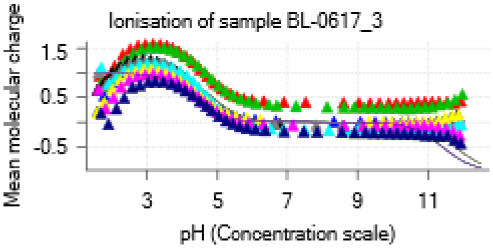

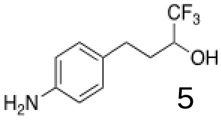

5

pH-metric Result

RMSD 8.861

Warnings and errors

Errors None
Warnings None

Graphs

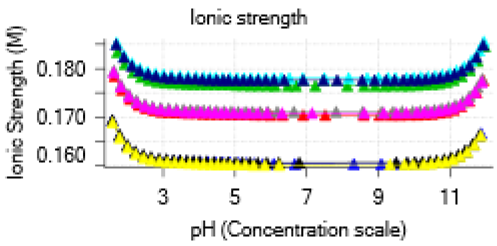

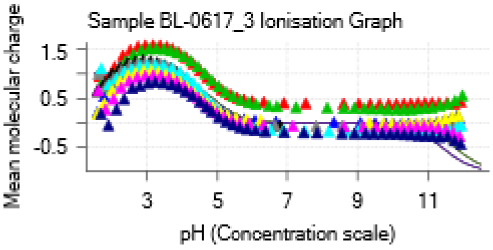

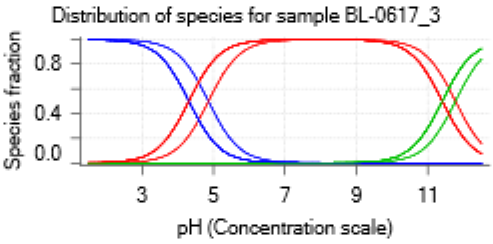

Multiset assays

Assay 1 of 3

Sample name	pH-metric psKa
Assay name	pH-metric psKa
Assay ID	18E-05002
Instrument ID	T317135
Imported from	C:\Sirius Data Backup\18E-05002_BL-0617_3_pH-metric psKa.t3r
Imported on	2/28/2023 7:11:11 PM
Analyst name	
Experiment start time	5/5/2018 8:36:56 AM

Assay 2 of 3

Sample name	pH-metric pKa
Assay name	pH-metric pKa
Assay ID	18E-04003

Multiset name:
Filename:

Instrument ID: **T317135**

pH-metric Result

logP (XH +) 0.00
logP (neutral X) 1.83
RMSD 33.017

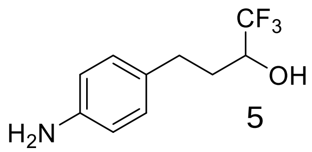

5

Warnings and errors

Errors None
Warnings None

Sample logD values

pH	BL-0617_3 logD	Comment
1.000	0.00	
1.200	0.01	Stomach pH
2.000	0.04	
3.000	0.27	
4.000	0.93	
5.000	1.58	
6.000	1.79	
6.500	1.82	
7.000	1.82	
7.400	1.83	Blood pH
8.000	1.83	
9.000	1.83	
10.000	1.83	
11.000	1.83	
12.000	1.83	

Graphs

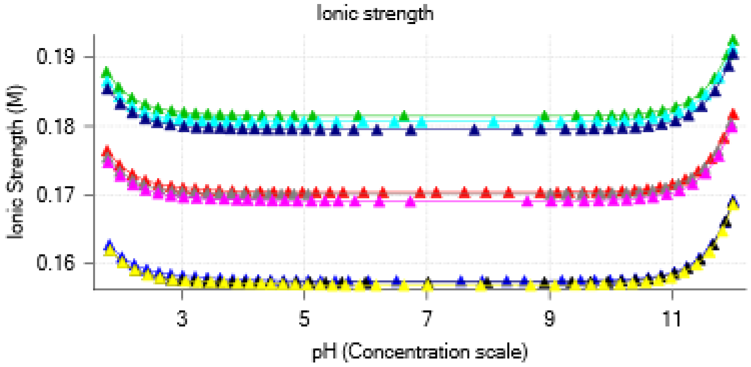

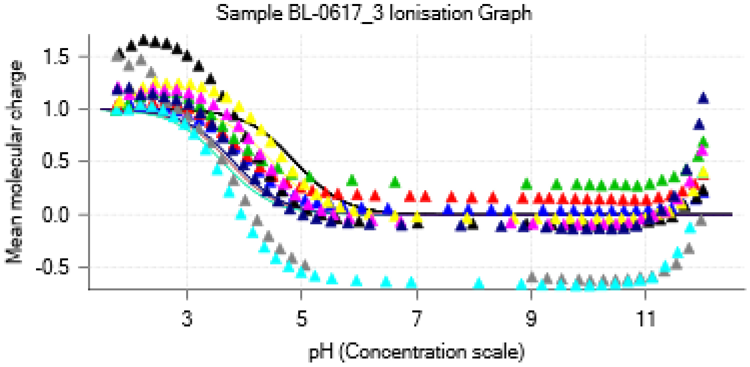

pH-metric Result

Multiset name: Instrument ID: **T317135**
Analyst: **CB**
Filename: **C:\SiriusData\2017\October\pKa_multiset_BL-0583.t3r**

pH-metric Result

Base pKa 1 4.87 ±0.00 (n=50)
RMSD 0.113

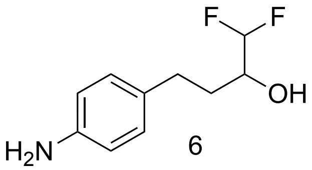

6

Warnings and errors

Errors None
Warnings None

Graphs

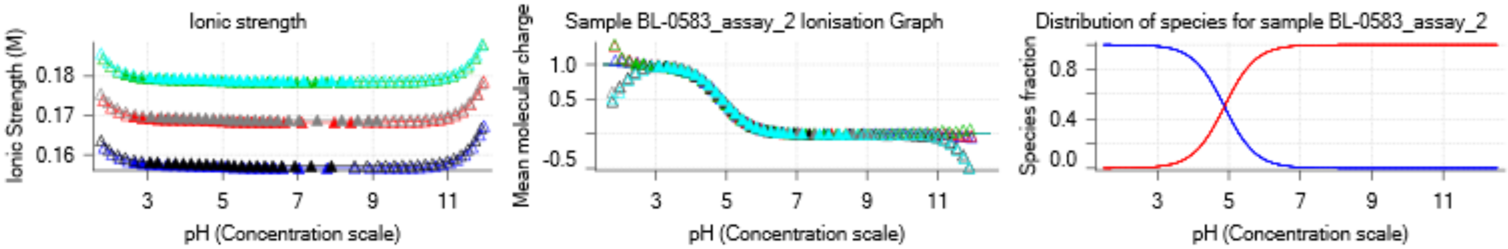

Multiset assays

Assay 1 of 2

Sample name	BL-0583_assay_2
Assay name	pH-metric pKa
Assay ID	17L-28003
Instrument ID	T317135
Imported from	C:\Users\SIRIUS\Desktop\Carlo\17L-28003_BL-0583_assay_2_BL-0583_assay_2.t3r
Imported on	12/28/2017 2:35:24 PM
Analyst name	CB
Experiment start time	12/28/2017 1:26:54 PM

Assay 2 of 2

Sample name	BL-0583
Assay name	pH-metric pKa
Assay ID	17L-20004
Instrument ID	T317135
Imported from	C:\Users\SIRIUS\Desktop\Carlo\17L-20004_BL-0583_BL-0583.t3r
Imported on	12/28/2017 2:35:24 PM
Analyst name	CB
Experiment start time	12/20/2017 5:27:54 PM

Notes

pH -metric determination of pKa. Multiset of two different experiments, each experiment included three different titrations. Experiment was done without co-solvent

pH-metric Result

Multiset name: Instrument ID: **T317135**

Analyst: **CB**

Quality: **Good**

Filename: **C:\SiriusData\2017\October\Multiset_logP_BL-0583.t3r**

pH-metric Result

logP (XH +) -1.04 ±0.04 (n=50)
logP (neutral X) 0.92 ±0.00 (n=50)
RMSD 0.197

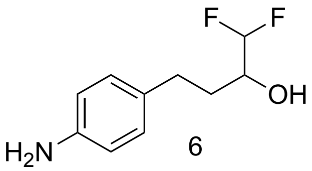

6

Warnings and errors

Errors None
Warnings None

Sample logD values

pH	BL-0583_assay_2 logD	Comment
1.000	-1.03	
1.200	-1.03	Stomach pH
2.000	-0.99	
3.000	-0.70	
4.000	0.03	
5.000	0.68	
6.000	0.89	
6.500	0.91	
7.000	0.92	
7.400	0.92	Blood pH
8.000	0.92	
9.000	0.92	
10.000	0.92	
11.000	0.92	
12.000	0.92	

Graphs

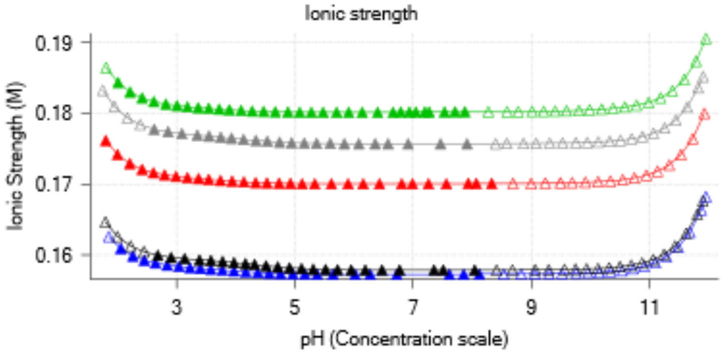

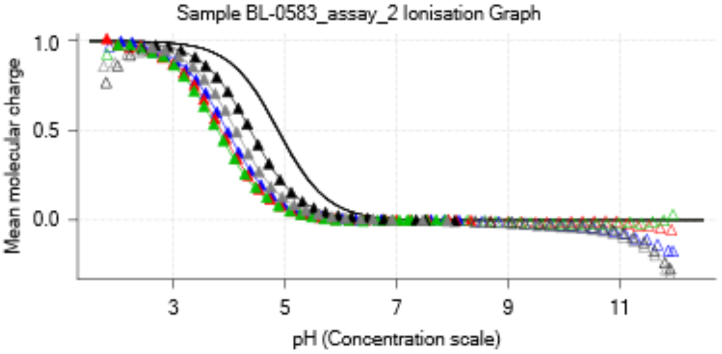

Yasuda-Shedlovsky Result

Multiset name:		Instrument ID:	**T317135**
Analyst:	**CB**		
Quality:	**Good**		
Filename:	**C:\Users\labuser\Desktop\Fluorinated isosteres\BL-0587 psKa multiset.t3r**		

Yasuda-Shedlovsky result

Extrapolation type	pKa 0%	SD	Intercept	Slope	R^2	Ionic strength	Temperature
Yasuda-Shedlovsky	4.69	±0.05	7.97	-120.8060	0.9704	0.163 M	25.0°C
Yasuda-Shedlovsky	8.85	±0.07	9.50	85.8998	0.8896	0.163 M	25.0°C

Warnings and errors

Errors None
Warnings None

Graphs

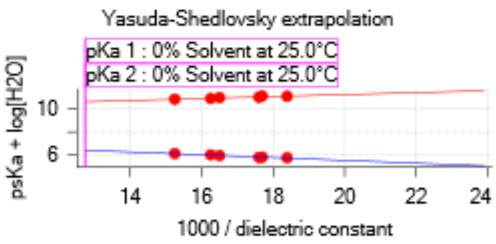

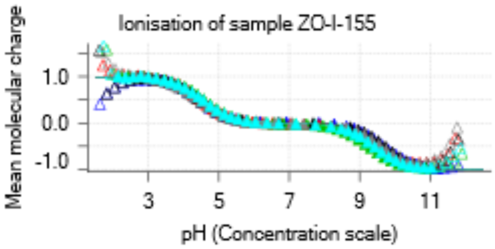

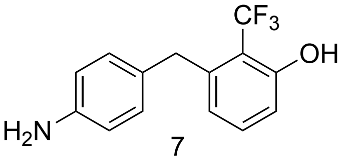

7

pH-metric Result

RMSD 0.096

Warnings and errors

Errors None
Warnings None

Graphs

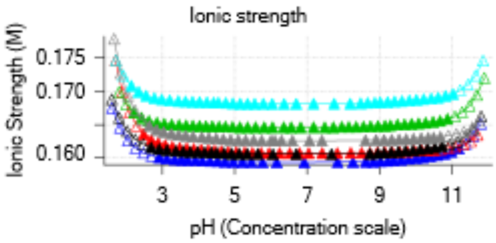

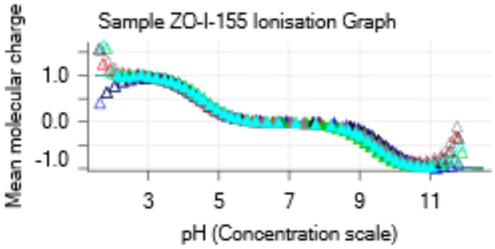

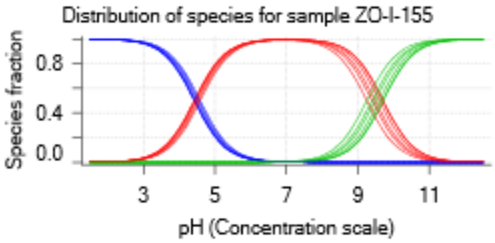

Multiset assays

Assay 1 of 2

Sample name	pKa_ZO-I-155_assay_2
Assay name	pH-metric psKa
Assay ID	18A-02004
Instrument ID	T317135
Imported from	C:\Users\SIRIUS\Desktop\Carlo\18A-02004_ZO-I-155_pKa_ZO-I-155_assay_2.t3r
Imported on	1/2/2018 1:20:46 PM
Analyst name	CB
Experiment start time	1/2/2018 12:08:30 PM

Assay 2 of 2

pH-metric Result

Multiset name:
Analyst: **CB**
Filename: **C:\Users\labuser\Desktop\Fluorinated isosteres\BL-0587 logP multiset.t3r**

Instrument ID: **T317135**

pH-metric Result

logP (XH2 +)	1.18	±0.09 (n=50)
logP (neutral XH)	3.18	±0.05 (n=50)
logP (X -)	-10.00	±0.35 (n=50)
RMSD	1.009	

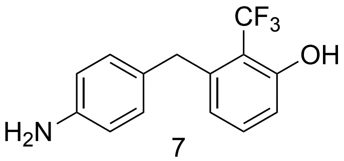

7

Warnings and errors

Errors None
Warnings None

Sample logD values

pH	ZO-I-155 logD	Comment
1.000	1.19	
1.200	1.19	Stomach pH
2.000	1.26	
3.000	1.66	
4.000	2.43	
5.000	3.01	
6.000	3.16	
6.500	3.17	
7.000	3.17	
7.400	3.17	Blood pH
8.000	3.12	
9.000	2.80	
10.000	2.00	
11.000	1.03	
12.000	0.03	

Graphs

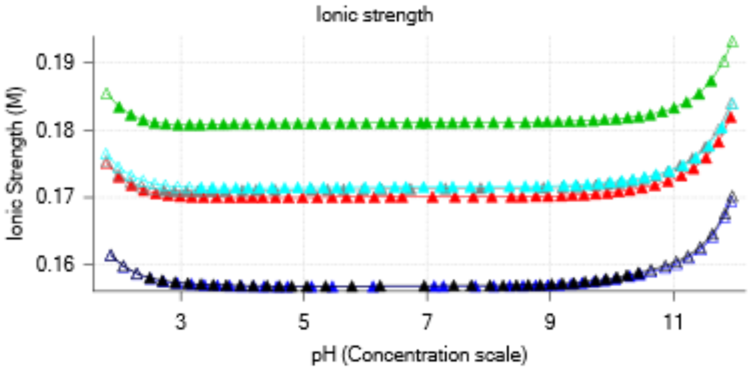

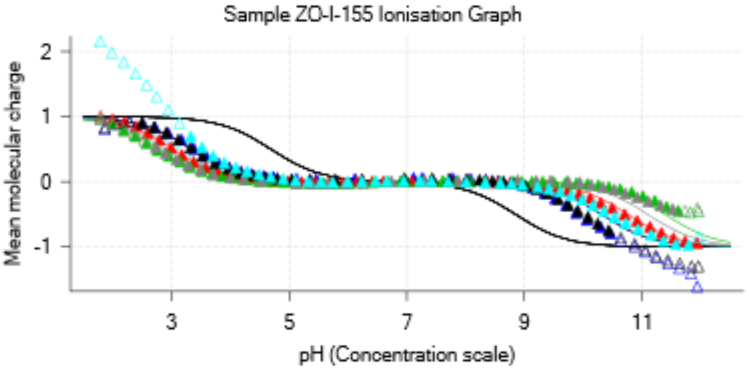

Multiset name:
Filename:

Instrument ID: **T317135**

pH-metric Result

Acid pKa 1	4.58	±0.03 (n=50)
Base pKa 2	9.67	±0.01 (n=50)
RMSD	0.389	

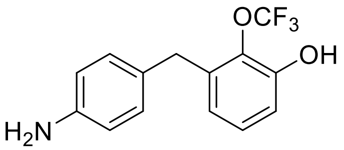

8

Warnings and errors

Errors	None
Warnings	None

Graphs

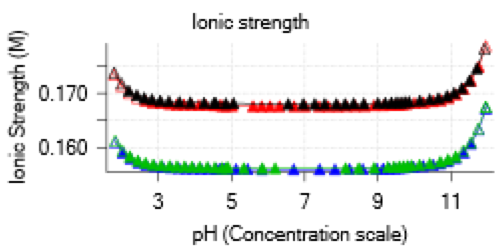

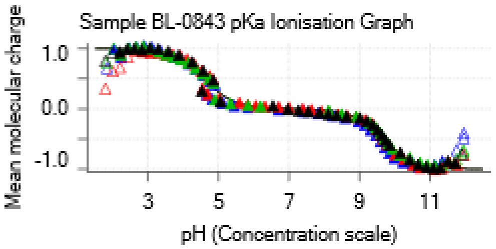

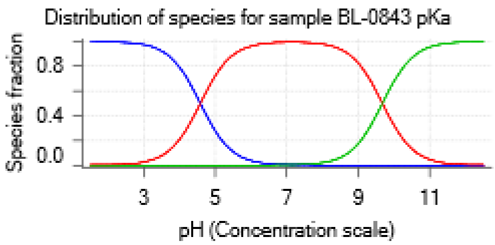

Multiset assays

Assay 1 of 2

Sample name	pH-metric pKa
Assay name	pH-metric pKa
Assay ID	21C-01002
Instrument ID	T317135
Imported from	C:\SiriusData\2021\Feb\21C-01002_BL-0843 pKa_pH-metric pKa.t3r
Imported on	3/1/2021 1:41:46 PM
Analyst name	
Experiment start time	3/1/2021 10:15:48 AM

Assay 2 of 2

Sample name	pH-metric pKa
Assay name	pH-metric pKa
Assay ID	21C-01003
Instrument ID	T317135
Imported from	C:\SiriusData\2021\Feb\21C-01003_BL-0843 pKa2_pH-metric pKa.t3r
Imported on	3/1/2021 1:41:49 PM
Analyst name	
Experiment start time	3/1/2021 11:39:56 AM

Sample name:
Assay name: **pH-metric medium logP**
Assay ID: **21C-01004**
Filename:

Experiment start time: **3/1/2021 1:55:33 PM**
Analyst:
Instrument ID: **T317135**

pH-metric Result

logP (XH2 +)	-4.43	±1.42 (n=50)
logP (neutral XH)	2.30	±0.05 (n=50)
logP (X -)	-10.00	
RMSD	1.607	

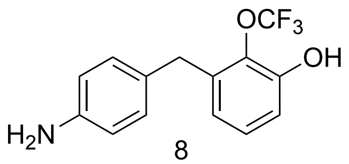

8

Warnings and errors

Errors None
Warnings None

Sample logD values

pH	logD	Comment
1.000	-1.28	
1.200	-1.08	Stomach pH
2.000	-0.28	
3.000	0.70	
4.000	1.61	
5.000	2.16	
6.000	2.28	
6.500	2.29	
7.000	2.29	
7.400	2.29	Blood pH
8.000	2.29	
9.000	2.21	
10.000	1.80	
11.000	0.95	
12.000	-0.04	

Graphs

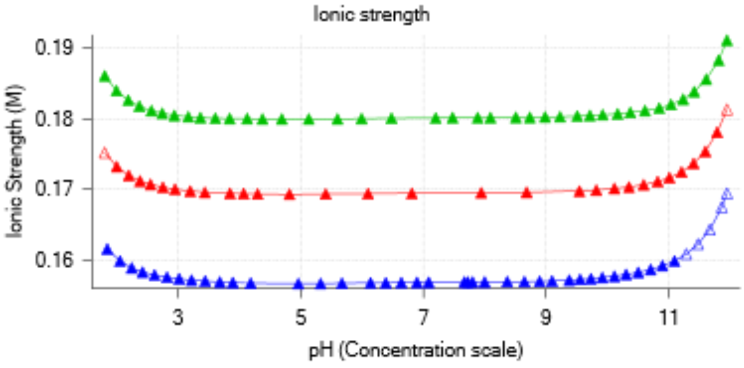

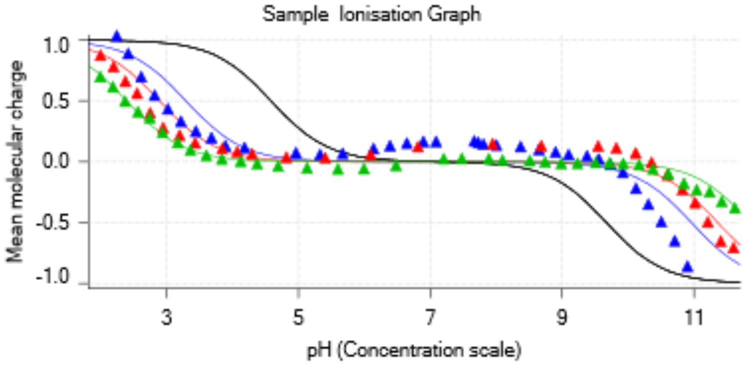

pH-metric Result

Multiset name:
Filename:

Instrument ID: **T317135**

pH-metric Result

Base pKa 1	4.24	±0.05 (n=50)
Acid pKa 2	9.51	±0.02 (n=50)
RMSD	0.502	

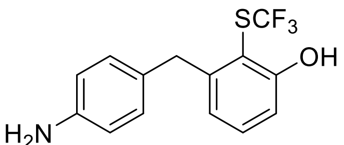

9

Warnings and errors

Errors	None
Warnings	None

Graphs

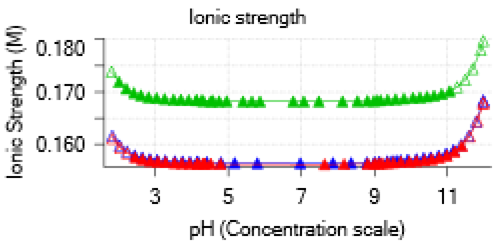

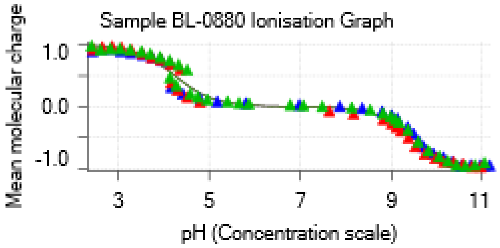

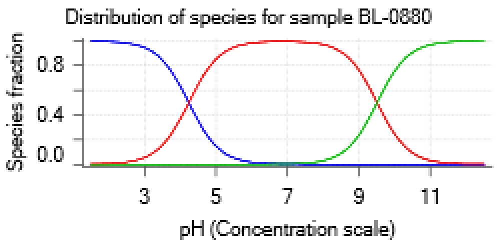

Multiset assays

Assay 1 of 2

Sample name	pH-metric pKa
Assay name	pH-metric pKa
Assay ID	21B-25003
Instrument ID	T317135
Imported from	C:\SiriusData\2021\Feb\21B-25003_BL-0880_pH-metric pKa.t3r
Imported on	2/26/2021 9:25:17 AM
Analyst name	
Experiment start time	2/25/2021 4:22:56 PM

Assay 2 of 2

Sample name	pH-metric pKa
Assay name	pH-metric pKa
Assay ID	21B-25004
Instrument ID	T317135
Imported from	C:\SiriusData\2021\Feb\21B-25004_BL-0880-2_pH-metric pKa.t3r
Imported on	2/26/2021 9:25:19 AM
Analyst name	
Experiment start time	2/25/2021 5:45:00 PM

pH-metric Result

Multiset name:
Filename: **C:\SiriusData\2021\Feb\BL-0880-logP.t3r**

Instrument ID: **T317135**

pH-metric Result

logP (XH2 +)	-8.93	±0.81 (n=48)
logP (neutral XH)	2.73	±0.01 (n=48)
logP (X -)	-10.00	
RMSD	0.928	

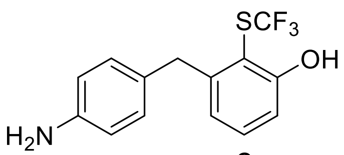

9

Warnings and errors

Errors	None
Warnings	None

Sample logD values

pH	BL-0880-logP2 logD	Comment
1.000	-0.51	
1.200	-0.31	Stomach pH
2.000	0.49	
3.000	1.46	
4.000	2.29	
5.000	2.66	
6.000	2.72	
6.500	2.73	
7.000	2.73	
7.400	2.72	Blood pH
8.000	2.71	
9.000	2.61	
10.000	2.12	
11.000	1.22	
12.000	0.24	

Graphs

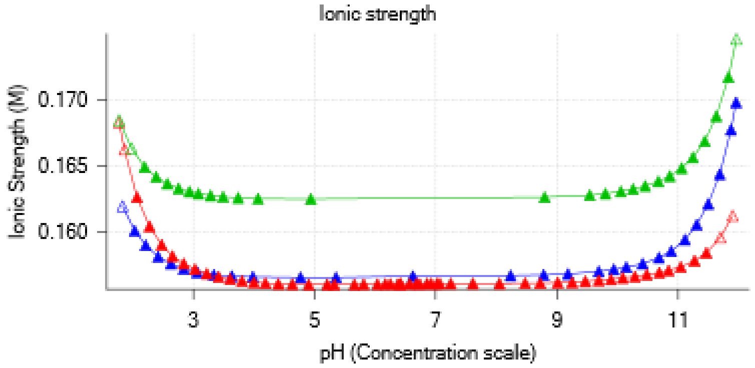

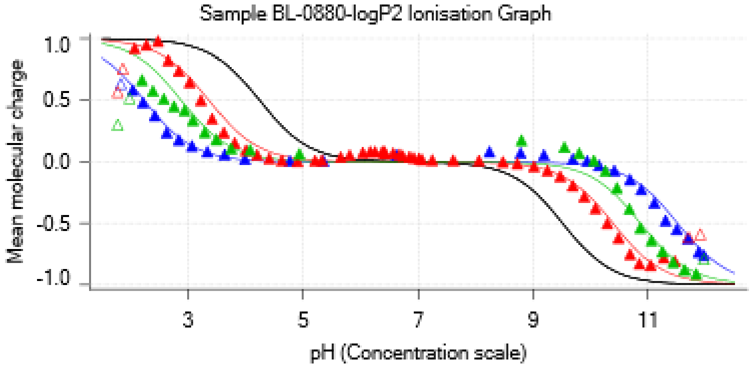

pH-metric Result

Multiset name: Instrument ID: T317135

Filename: **C:\SiriusData\2022\April 2022\December 2022\22L-06004_BL-0949 multiset-pH-metric pKa.t3r**

pH-metric Result

Acid pKa 1 4.21 ±0.01 (n=50)
Base pKa 2 7.93 ±0.03 (n=50)
RMSD 0.058

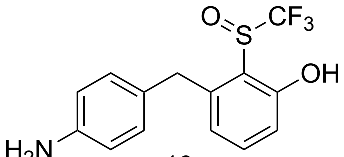

10

Warnings and errors

Errors None
Warnings None

Graphs

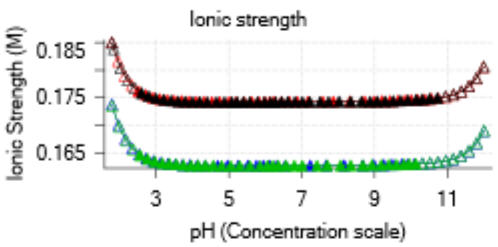

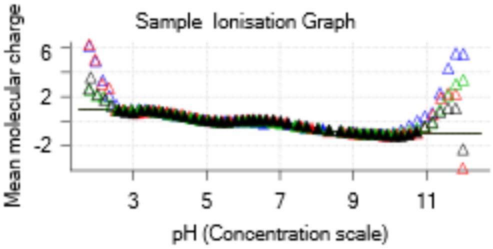

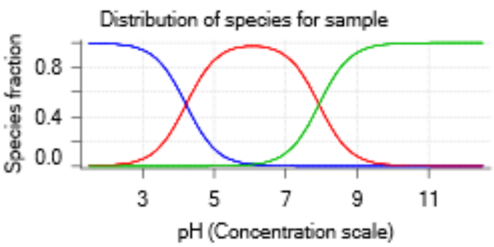

Multiset assays

Assay 1 of 2

Sample name	BL-0949-pH-metric pKa
Assay name	pH-metric pKa
Assay ID	22L-06004
Instrument ID	T317135
Imported from	C:\SiriusData\2022\April 2022\December 2022\22L-06004_BL-0949-pH-metric pKa.t3r
Imported on	12/6/2022 6:43:26 PM
Analyst name	
Experiment start time	12/6/2022 4:32:11 PM

Assay 2 of 2

Sample name	BL-0949b-pH-metric pKa
Assay name	pH-metric pKa
Assay ID	22L-06005
Instrument ID	T317135
Imported from	C:\SiriusData\2022\April 2022\December 2022\22L-06005_BL-0949b-pH-metric pKa.t3r
Imported on	12/6/2022 6:43:27 PM
Analyst name	
Experiment start time	12/6/2022 5:48:21 PM

Multiset name:
Filename:

Instrument ID: **T317135**

pH-metric Result

logP (neutral XH2)	5.58
logP (XH -)	0.51
logP (X 2-)	-10.00
RMSD	1.020

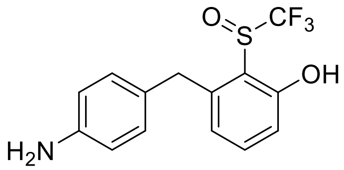

10

Assay 22L-08002 - BL-0949b-LogP

Carbonate	0.0000 mM
Acidity error	0.00000 mM

Warnings and errors

Errors	None
Warnings	Excessive carbonate concentration present

Sample logD values

pH	logD	Comment
1.000	5.58	
1.200	5.58	Stomach pH
2.000	5.57	
3.000	5.51	
4.000	5.15	
5.000	4.33	
6.000	3.35	
6.500	2.85	
7.000	2.36	
7.400	1.96	Blood pH
8.000	1.39	
9.000	0.61	
10.000	-0.12	
11.000	-1.06	
12.000	-2.05	

Graphs

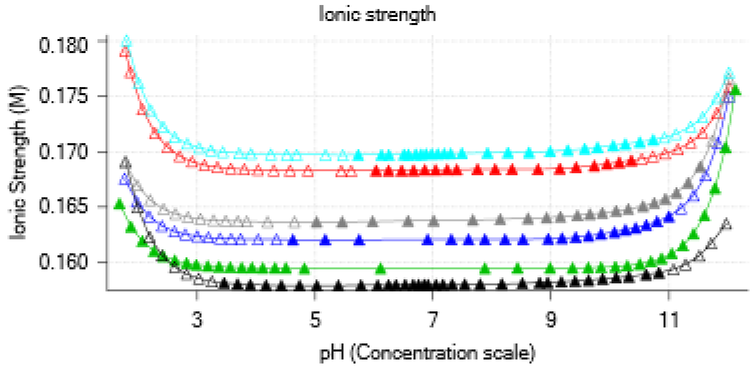

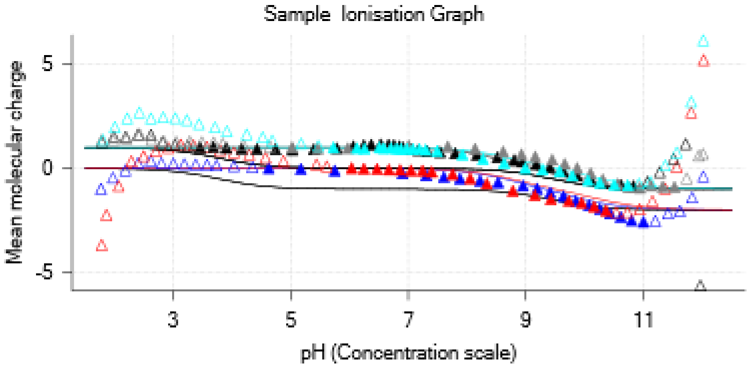

Yasuda-Shedlovsky Result

Sample name:	**BL-0544.sdf**	Authentication:	**Failed**
Assay name:	**pH-metric psKa**	Experiment start time:	**10/19/2017 12:12:41 PM**
Assay ID:	**17J-19005**	Analyst:	**CB**
Quality:	**Good**	Instrument ID:	**T317135**
Filename:	**C:\SiriusData\2017\October\17J-19005_BL-0544 (ZO-I-104)_pH-metric psKa.t3r**		

Yasuda-Shedlovsky result

Extrapolation type	pKa 0%	SD	Intercept	Slope	R^2	Ionic strength	Temperature
Yasuda-Shedlovsky	8.91	±0.04	9.76	70.1947	0.9872	0.164 M	25.0°C

Warnings and errors

Errors	None
Warnings	None

Graphs

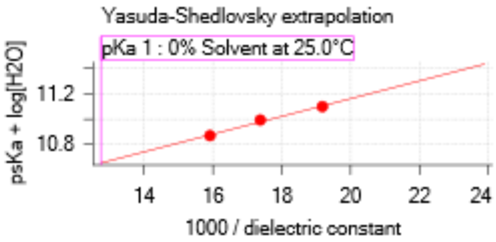

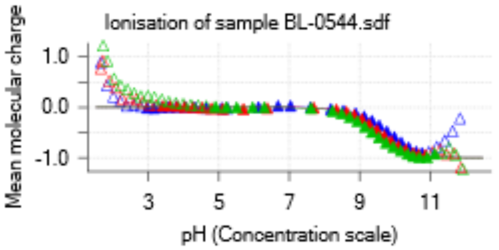

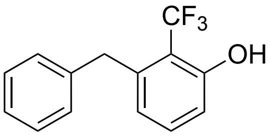

32

pH-metric Result

RMSD 0.188

Warnings and errors

Errors	None
Warnings	None

Graphs

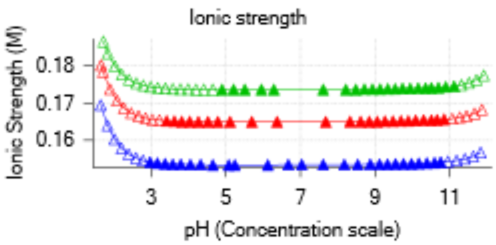

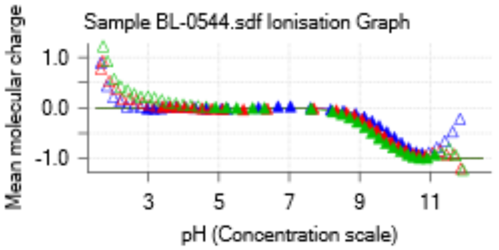

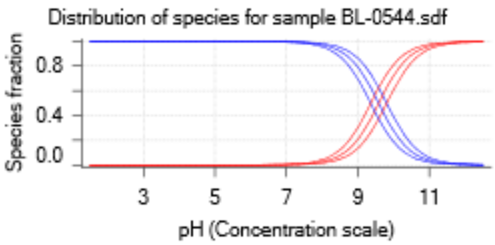

pH-metric Result

Sample name:	**BL-0544 LogP**	Experiment start time:	**5/20/2021 6:11:05 PM**
Assay name:	**pH-metric high logP**	Analyst:	
Assay ID:	**21E-20004**	Instrument ID:	**T317135**
Filename:	**C:\SiriusData\2021\May\21E-20004_BL-0544 LogP_pH-metric high logP.t3r**		

pH-metric Result

logP (neutral XH)	4.32
logP (X -)	-7.03
RMSD	0.486

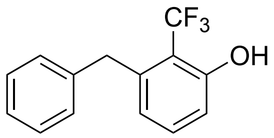

32

Warnings and errors

Errors	None
Warnings	None

Sample logD values

**pH**	**BL-0544 LogP logD**	**Comment**
1.000	4.32	
1.200	4.32	Stomach pH
2.000	4.32	
3.000	4.32	
4.000	4.32	
5.000	4.32	
6.000	4.32	
6.500	4.32	
7.000	4.31	
7.400	4.29	Blood pH
8.000	4.21	
9.000	3.74	
10.000	2.86	
11.000	1.87	
12.000	0.87	

Graphs

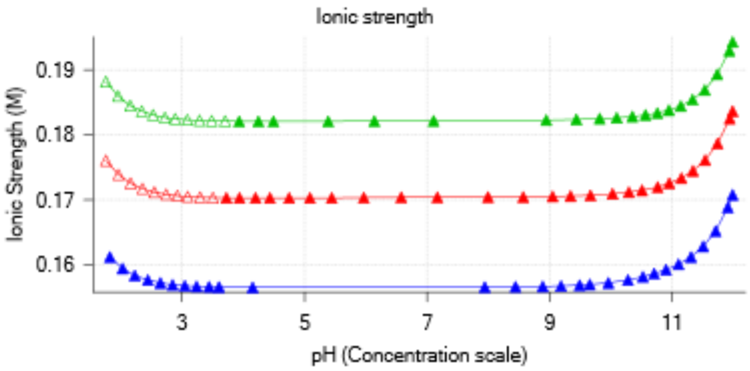

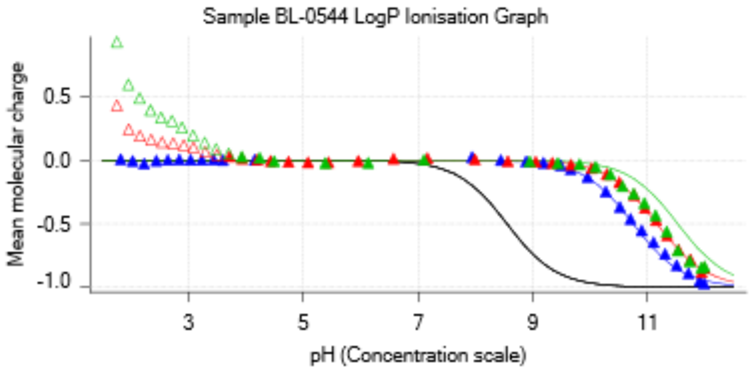

Supplement: Supporting Info2 [file NIHMS1980772-supplement-Supporting_Info2.pdf]
